# Robust joint clustering of multi-omics single-cell data via multi-modal high-order neighborhood Laplacian matrix optimization

**DOI:** 10.1093/bioinformatics/btad414

**Published:** 2023-06-29

**Authors:** Hao Jiang, Senwen Zhan, Wai-Ki Ching, Luonan Chen

**Affiliations:** School of Mathematics, Renmin University of China, Beijing 100872, China; School of Mathematics, Renmin University of China, Beijing 100872, China; Department of Mathematics, The University of Hong Kong, Pokfulam Road, Hong Kong; Key Laboratory of Systems Biology, Shanghai Institute of Biochemistry and Cell Biology, CAS Center for Excellence in Molecular Cell Science, Chinese Academy of Sciences, Shanghai 200031, China; Key Laboratory of Systems Health Science of Zhejiang Province, School of Life Science, Hangzhou Institute for Advanced Study, University of Chinese Academy of Sciences, Chinese Academy of Sciences, Hangzhou 310024, China

## Abstract

**Motivation:**

Simultaneous profiling of multi-omics single-cell data represents exciting technological advancements for understanding cellular states and heterogeneity. Cellular indexing of transcriptomes and epitopes by sequencing allowed for parallel quantification of cell-surface protein expression and transcriptome profiling in the same cells; methylome and transcriptome sequencing from single cells allows for analysis of transcriptomic and epigenomic profiling in the same individual cells. However, effective integration method for mining the heterogeneity of cells over the noisy, sparse, and complex multi-modal data is in growing need.

**Results:**

In this article, we propose a multi-modal high-order neighborhood Laplacian matrix optimization framework for integrating the multi-omics single-cell data: scHoML. Hierarchical clustering method was presented for analyzing the optimal embedding representation and identifying cell clusters in a robust manner. This novel method by integrating high-order and multi-modal Laplacian matrices would robustly represent the complex data structures and allow for systematic analysis at the multi-omics single-cell level, thus promoting further biological discoveries.

**Availability and implementation:**

Matlab code is available at https://github.com/jianghruc/scHoML.

## 1 Introduction

With the first release of single-cell transcriptome analysis technology in 2009 (Tang *et al.* 2009), an explosion of research has been conducted in obtaining high-resolution views of single-cell RNA-seq data, such as Smart-seq (Ramsköld *et al.* 2012) and Smart-seq2 (Picelli *et al.* 2014); *in vitro* transcription-based Cel-seq (Hashimshony *et al.* 2012) and Cel-seq2 (Hashimshony *et al.* 2016); and designed primer-based MALBAC (Zong *et al.* 2012), etc. Advances in scRNA-seq technologies have enabled the exploration of cellular heterogeneity where traditional bulk sequencing cannot reveal. In Zhang *et al.* (2018), T cell heterogeneity was investigated in colorectal cancer. scRNA-seq data were introduced for analyzing genetic tumor heterogeneity in Fan *et al.* (2018). Intra-tumoral heterogeneity of pancreatic ductal adenocarcinoma was highlighted in Peng (2019). In Sorek *et al.* (2021), transcriptional heterogeneity was discovered in disease-state neurons. Ren *et al.* (2021) applied scRNA-seq to obtain comprehensive immune landscape for better understanding of COVID-19.

Apart from biological research in heterogeneity analysis using scRNA-seq techniques, extensive research has been carried out in developing effective and efficient computational methods for exploring cellular heterogeneity. For instance, cell-pair differentiability correlation was proposed in evaluating cellular relationships (Jiang *et al.* 2018), and further incorporated for cellular heterogeneity analysis. Semi-supervised clustering method (Chen *et al.* 2021) was developed for analyzing scRNA-seq data. When data are in large scale, efficient hierarchical clustering algorithm was developed (Zou *et al.* 2021). Also, method focused on similarity learning was proposed in Mei *et al.* (2021) for identifying cell types using scRNA-seq data. Recent progress has shed light on graph attention auto-encoder for scRNA-seq data representation and clustering (Cheng and Ma 2022).

Cell state, as usually evaluated by RNA expression, may not fully capture the complex structure embedded. It is a complex representation determined by the interplay between transcriptome, proteome, epigenome, etc. Multi-omics single-cell sequencing technologies, by profiling multiple types of “omics” expression in the same individual cells, enable the exploration of cellular heterogeneity in an integrative way. Cellular indexing of transcriptomes and epitopes by sequencing (CITE-seq) can simultaneously measure RNA expression and surface protein abundance via antibody-derived tags, and robust protein profiling contribute for better understanding of cell states (Stoeckius *et al.* 2017). Single-cell methylation and transcriptome sequencing (scM&T-seq) technique allows for quantification of transcriptomic and epigenomic expression in the same individual cells (Angermueller 2016). Different “omics” layers will together present more accurate and complete view of “single cell state” and enable dissecting regulatory heterogeneity from complex cell populations.

With the flourishing development of single-cell multi-omics technologies, a growing number of methods have been proposed for integrating multi-omics data. These methods can be categorized into two major types. A major type of methods was designed for multi-omics data measured in different cells. Representative methods include Seurat (Stuart *et al.* 2019) for integrating scRNA-seq and scATAC-seq data. LIGER (Welch *et al.* 2019) was developed to align scRNA-seq data and single-cell epigenomic data in a low-dimensional space. MATCHER (Welch *et al.* 2017) utilized a Gaussian process latent variable model to evaluate the correlations between scRNA-seq data and single-cell epigenomic measurements from different cells. In Zhana *et al.* (2018), a coupled nonnegative matrix factorization method was proposed for integrating scRNA-seq and scATAC-seq data. Another type of methods was proposed for integration of multi-omics profiles measured in the same set of cells. scAI (Jin 2020) proposed a regularized matrix factorization framework to iteratively learn a low-dimensional representation for the multi-modal single-cell data. In BREM-SC (Wang *et al.* 2020), a Bayesian random effects mixture model was developed for joint clustering single-cell transcriptomic and proteomic data generated by CITE-seq, where multinomial distribution was proposed to model scRNA-seq data and Dirichlet multinomial distribution was proposed to model surface protein (ADT) data. Hao *et al.* (2021) designed a weighted nearest-neighbor framework for integrating multi-modal single-cell data. An elegant design of modal weights in generating the weighted combination of modality affinities is proposed to measure the final weighted similarity metric integrating multiple modalities of single-cell data. It would be an interesting topic on adaptively and automatically determining a weighted combination of modal-specific “similarities” or “affinities.” Considering the inherent relationship between chromatin accessibility and gene expression, Duren *et al.* (2022) proposed a new concept: cis-regulatory potential to formulate a matrix-factorization framework to integrate scRNA-seq data and scATAC-seq data. scAB (Zhang *et al.* 2022) integrated scRNA-seq data or scATAC-seq data with annotated bulk sequencing data incorporating knowledge and guided graph information. The phenotype-associated cell states and signatures were elucidated through matrix factorization framework on the Pearson correlation matrix linking single-cell data and bulk RNA sequencing data. There are also attempts in using deep learning frameworks for single-cell multi-omics data integration such as GLUE (Cao and Gao 2022), a knowledge-based guidance graph-linked unified embedding method using variational autoencoders. The incorporation of knowledge either from gene–gene interaction network or bulk sequencing data would positively contribute to a better understanding of the cell states and characteristics described by single-cell omics data. However, the generalization ability in other multi-omics integration may be constrained. The development of integration techniques for single-cell multi-omics data without knowledge information is in growing need as well.

In this article, we focus on integrative analysis of parallel multi-omics single-cell data. Most of the current methods mainly aim to model a common low-dimensional embedding or unified relationship between cells. Taking into consideration of the sparsity and nonlinearity nature of single-cell data, also inspired by the above findings, we propose a multi-modal high-order neighborhood Laplacian matrix optimization framework (scHoML) for integrating multi-omics single-cell data. The method is very flexible and robust, which can be applied for efficiently integrating multi-omics data both in simulation and real-world datasets generated by scM &T-seq, CITE-seq technologies, etc. The article is structured as follows. In Section 2, we present preliminary information on the framework for multi-omics data integration. Section 3 presents the method scHoML for integrating multi-omics data. Experimental results are presented in Section 4. Section 5 discusses the application capability of scHoML. Finally, Section 6 concludes the article.

## 2 Preliminaries

In this section, we provide preliminary information on the framework for multi-omics data integration.

- **High-order networks**

Assume single modal dataset $X={\left[x_{1},x_{2},\ldots ,x_{n}\right]}^{T}\in R^{n\times d}$, where *n* is the number of samples, *d* is the dimensionality of the attributes. We first present the high-order networks for the dataset *X* modeling the connectivity of the data through definitions of adjacent matrices in different orders $W_{i},\;i=1,2\ldots ,U$.

In the construction of the first-order adjacent matrix *W*_1_, where $A_{jk}=\text{exp}\;\left(-\frac{||x_{j}-x_{k}|{|}^{2}}{2\sigma^{2}}\right),\;1\leq j,k\leq n.$ Here, a pair of vertices (*j*, *k*) is connected if and only if vertex *j* is in the nearest neighbors of the vertex *k*.


(1) $$w_{1,jk}=\{\begin{array}{cc} A_{jk}, & \text{if}\;a\;\text{pair}\;\text{of}\;\text{vertices}\;(j,k)\;\text{is}\;\text{connected}\\ 0, & \text{otherwise} \end{array}$$

In the construction of high-order adjacent matrix *W_i_*, we follow the similar concept in obtaining $W_{i-1}$. If $w_{i-1,j}$ is similar to $w_{i-1,k}$, vertex *j* is also similar to vertex *k* in a *i −* 1th-order connectivity. Let $w_{i,j}=(w_{i,j1},w_{i,j2},\ldots ,w_{i,jn})$ represent the *j*th row of *W_i_*, high-order adjacent matrix $W_{i},\;i\geq 2$ can be derived in the following formulation. where $w_{i,jj}=0,\;j=1,2,\ldots ,n$, making *W_i_* satisfy the form of adjacent matrix.


(2) $$w_{i,jk}=\{\begin{array}{cc} \;\text{exp}\;\left(-\frac{||w_{i-1,j}-w_{i-1,k}|{|}^{2}}{2\sigma^{2}}\right), & j\neq k,\;\text{if}\;\exists t,\;s.t.\\ & w_{i-1,jt}>0,\;w_{i-1,kt}>0\\ 0, & \text{otherwise} \end{array}$$

- **High-order Laplacian matrix**

The corresponding normalized Laplacian matrices in different orders can be derived based on $W_{i},\;i=1,2,3,\ldots$.

Let $D_{i}\in R^{n\times n}$ represent its *i*th-order degree matrix, a diagonal matrix and $D_{i,jj}=\sum_{k=1}^{n}w_{i,jk}$ The *i*th-order normalized Laplacian matrix can be defined as


$$L^{\left(i\right)}=I_{n}-{\left(D_{i}\right)}^{-\frac{1}{2}}W_{i}{\left(D_{i}\right)}^{-\frac{1}{2}},\;i=1,2,\ldots$$

- **Multi-modal Laplacian matrix**

Suppose we have paralleled profiled single-cell multi-omics data in *V* modals $X^{\left(1\right)},X^{\left(2\right)},\ldots ,X^{\left(V\right)}$, where $X^{\left(i\right)}\in R^{n\times d^{\left(i\right)}},\;i=1,2,\ldots ,V$ is the dataset of *i*th modal and $d^{\left(i\right)}$ represents the attribute dimensionality of *i*th modal.

According to the steps in construction of high-order networks and high-order Laplacian matrix, we define $L_{p}^{\left(i\right)},i=1,2,\ldots ,U$ to represent the *i*th-order normalized Laplacian matrix for the *p*th modal, i.e. $L_{p}^{\left(i\right)}=I_{n}-{\left(D_{p}^{\left(i\right)}\right)}^{-\frac{1}{2}}W_{p}^{\left(i\right)}{\left(D_{p}^{\left(i\right)}\right)}^{-\frac{1}{2}},\;p=1,2,\ldots ,V$, where $W_{p}^{\left(i\right)}\in R^{n\times n}$ represent *i*th-order adjacent matrix for *p*th modal single-cell data, $D_{p}^{\left(i\right)}\in R^{n\times n}$ represent *i*th-order degree matrix for the *p*th modal single-cell data.

Hence, in single-cell data composed of *V* modals, we have in total *U *×* V* Laplacian matrices for different modals and different orders.

- **Multi-modal multi-order Laplacian matrix fusion**

In multi-modal single-cell data integration, how to integrate the modal-specific Laplacian matrices to formulate a fused, appropriate Laplacian matrix is a central and critical problem.

Taking into consideration on the modal-specific Laplacian matrices, we first integrate Laplacian matrices for specific order *i*, i=1,2,…,U, where to integrate modal-specific proximity information from multi-modal data.


$$L_{\mu }^{\left(i\right)}=\sum_{p=1}^{V}\mu_{p}L_{p}^{\left(i\right)},\;\sum_{p=1}^{V}\mu_{p}=1,\;\mu_{p}\geq 0,\;p=1,2,\ldots ,V,$$

Second, incorporating high-order connectivity information embedded in the high-order Laplacian matrix, we propose the linear combination of $L_{\mu }^{\left(i\right)},\;i=1,2,\ldots ,U$ to formulate the fused Laplacian matrix for multi-omics single-cell data


(3) $$L^{*}=\sum_{i=1}^{U}\lambda_{i}L_{\mu }^{\left(i\right)},\;\sum_{i=1}^{U}\lambda_{i}=1,\;\lambda_{i}\geq 0,\;i=1,2,\ldots ,U.$$

Intuitively, we aim to approximate the fused Laplacian matrix $L^{*}$ as the linear combination of different order Laplacian matrix $L_{\mu }^{\left(i\right)},i=1,2,\ldots ,U$, respectively, to incorporate comprehensive structure information and seek better representation capability of the relationship described by multi-modal data.

## 3 Materials and methods

In this section, we present the method for integrating multi-omics data, to optimize the fused Laplacian matrix, as well as obtaining the low-dimensional representation for the multi-omics data.

For single modal data, spectral clustering can be realized through solving optimization problem with given Laplacian matrix *L*. In terms of multi-omics single-cell data, the proposed Laplacian matrix $L^{*}$ approximated by linear combination of different order modal-specific Laplacian matrices in the form $\sum_{i=1}^{U}\lambda_{i}L_{\mu }^{\left(i\right)}$ has several parameters yet to be determined.

How to automatically determine the parameters embedded in Laplacian matrix $L^{*}$ and seek better representation capability of the common embedding for multi-modal data thus constitutes a critical challenge. Motivated by the framework of spectral clustering, we propose the optimization objective as minimization of $\text{tr}(H^{T}L^{*}H)$, while simultaneously seek optimized *H* as the low-dimensional embedding for the multi-modal data, and the optimization problem can be expressed as follows:


(4) $$\begin{array}{c} \underset{\lambda ,H,\mu }{\min}\;\text{tr}(H^{T}L^{*}H)+{\left\|L^{*}-\sum_{i=1}^{U}\lambda_{i}L_{\mu }^{\left(i\right)}\right\|}_{F}^{2}\\ s.t.\;L_{\mu }^{\left(i\right)}=\sum_{p=1}^{V}\mu_{p}L_{p}^{\left(i\right)}\;(i=1,2,\ldots ,U),\\ L^{*}\;(\text{positive-semi-definite}),\;L_{jk}^{*}\leq 0,\;j\neq k\\ H\in R^{n\times c},\;H^{T}H=I_{c}\\ \mu ={\left[\mu_{1},\mu_{2},\ldots ,\mu_{V}\right]}^{T},\;||\mu |{|}_{1}=1,\;\mu \geq 0\\ \lambda ={\left[\lambda_{1},\lambda_{2},\ldots ,\lambda_{U}\right]}^{T},\;||\lambda |{|}_{1}=1,\;\lambda \geq 0. \end{array}$$

### 3.1 Multi-modal Laplacian matrix optimization

High-order Laplacian matrix can model the hidden high-order connection information among data, but the value of order needs properly selected as too high order may distort the original relationship embedded in the dataset. Hence, we focus on integration of Laplacian matrix in first order and second order, to preserve global data structure in a better manner, as well as improving learning performance. However, the positive-semi-definite property of $L^{*}$ added in the constraints of the optimization problem makes the optimization problem hard and inefficient to solve. Taking into consideration on the original definition of Laplacian matrix $I_{n}-D^{-1/2}W_{1}D^{-1/2}$, and the symmetric property of *W*_1_ that can be decomposed into eigen-matrix form $W_{1}=\tilde{U}\Lambda {\tilde{U}}^{T}$, the optimization term $L^{*}$ can be reformulated with $I_{n}-W\Lambda W^{T}$, hence we alternatively propose the final optimization problem in the following:


(5) $$\begin{array}{c} \underset{\lambda ,W,\Lambda ,H,\mu }{\min}\;\text{tr}(H^{T}(I_{n}-W\Lambda W^{T})H)\\ +{\left\|I_{n}-W\Lambda W^{T}-(\lambda L_{\mu }^{\left(1\right)}+(1-\lambda )L_{\mu }^{\left(2\right)})\right\|}_{F}^{2}\\ s.t.\;L_{\mu }^{\left(i\right)}=\sum_{p=1}^{V}\mu_{p}L_{p}^{\left(i\right)}\;(i=1,2),\\ W,H\in R^{n\times c},\;W^{T}W=I_{c},\;H^{T}H=I_{c}\\ \mu ={\left[\mu_{1},\mu_{2},\ldots ,\mu_{V}\right]}^{T},\;||\mu |{|}_{1}=1,\;\mu \geq 0\\ 0\leq \Lambda_{kk}\leq 1,\;k=1,2,\ldots ,c,\;0\leq \lambda \leq 1. \end{array}$$

Here Λ is a diagonal matrix, and $0\leq \Lambda_{kk}\leq 1$ makes sure the optimization stable.

#### 3.1.1 Optimization framework

Taking into consideration on the nonconvexity of the above problem, we propose alternative optimization framework to solve the problem by updating each variable iteratively. For the convenience of optimization, we rewrite $||I_{n}-W\Lambda W^{T}-(\lambda L_{\mu }^{\left(1\right)}+(1-\lambda )L_{\mu }^{\left(2\right)})|{|}_{F}^{2}$ into the following form:


$$\begin{array}{c} \text{tr}[I_{n}-2W\Lambda W^{T}-2(\lambda L_{\mu }^{\left(1\right)}+(1-\lambda )L_{\mu }^{\left(2\right)})\\ +2W\Lambda W^{T}(\lambda L_{\mu }^{\left(1\right)}+(1-\lambda )L_{\mu }^{\left(2\right)})\\ +W\Lambda^{2}W^{T}+{\left(\lambda L_{\mu }^{\left(1\right)}+(1-\lambda )L_{\mu }^{\left(2\right)}\right)}^{2}]. \end{array}$$

The optimization process consists of the following five steps:

- *Updating* λ: Fixing W,Λ,H,μ, the update of λ can be realized through solving the optimization problem:
  (6) $$\begin{array}{c} \underset{0\leq \lambda \leq 1}{\min}\;\text{tr}\left[{\left(\lambda L_{\mu }^{\left(1\right)}+(1-\lambda )L_{\mu }^{\left(2\right)}\right)}^{2}\right]\\ -2\text{tr}\left[\left(I_{n}-W\Lambda W^{T})(\lambda L_{\mu }^{\left(1\right)}+(1-\lambda )L_{\mu }^{\left(2\right)}\right)\right] \end{array}$$
  Define
  $$\begin{array}{c} a=\text{tr}\left[{\left(L_{\mu }^{\left(1\right)}\right)}^{2}-2L_{\mu }^{\left(1\right)}L_{\mu }^{\left(2\right)}+{\left(L_{\mu }^{\left(2\right)}\right)}^{2}\right]=||L_{\mu }^{\left(1\right)}-L_{\mu }^{\left(2\right)}|{|}_{F}^{2}\geq 0.\\ b=2\text{tr}\left[\left(L_{\mu }^{\left(2\right)}-I_{n}+W\Lambda W^{T})(L_{\mu }^{\left(1\right)}-L_{\mu }^{\left(2\right)}\right)\right]. \end{array}$$
  1. If *a* > 0, we can obtain
    $$\begin{array}{c} \lambda =\{\begin{array}{cc} 0, & \text{if}-\frac{b}{2a}<0\\ 1, & \text{if}-\frac{b}{2a}>1\\ -\frac{b}{2a}, & \text{otherwise} \end{array} \end{array}$$
- If *a* = 0, then we can deduce *b* = 0. In this case, we keep *λ* unchanged. That means if *λ_k_* represent the value of *λ* in *k*th iteration, we will let $\lambda_{k}=\lambda_{k-1}$.

- *Updating* ***W***: Given fixed λ,Λ,H,μ, the update of *W* can be generated through the optimization problem as follows:
  (7) $$\underset{W^{I}W=I_{c}}{\min}\;\text{tr}(\Lambda W^{T}BW)$$
  where $B=\lambda L_{\mu }^{\left(1\right)}+(1-\lambda )L_{\mu }^{\left(2\right)}-\frac{1}{2}HH^{T}$.
  The solution *W* of Equation (7) can be calculated as the first *c* eigenvectors of *B* (Zhou *et al.* 2020).
- *Updating* Λ: Given fixed λ,W,H,μ, we optimize the following problem to update Λ:
  (8) $$\begin{array}{c} \underset{0\leq \Lambda_{ii}\leq 1,\Lambda_{ij}=0(i\neq j)}{\min}\;\text{tr}(\Lambda^{2}+2\Lambda C), \end{array}$$
  where
  $$C=W^{T}\left[(\lambda L_{\mu }^{\left(1\right)}+(1-\lambda )L_{\mu }^{\left(2\right)})-\frac{1}{2}HH^{T}\right]W-I_{c}$$
  We can get:
  $$\begin{array}{c} \Lambda_{ii}=\{\begin{array}{cc} 0, & C_{ii}\geq 0\\ 1, & C_{ii}\leq -1\\ -C_{ii}, & \text{otherwise} \end{array} \end{array}$$
- *Updating* ***H***: Fixing λ,W,Λ,μ, the optimization problem with respect to *H* can be reduced into the following formula:
  (9) $$\underset{H^{T}H=I_{c}}{\min}\;\text{tr}(H^{T}(I_{n}-W\Lambda W^{T})H)$$
  Then we can obtain the solution *H* of Equation (9) by calculating the first *c* eigenvectors of $I_{n}-W\Lambda W^{T}$.
- *Updating* μ: Given fixed λ,W,Λ,H, then we can optimize the problem in the following form:
  (10) $$\begin{array}{c} \underset{||\mu |{|}_{1}=1,\mu \geq 0}{\min}\;\text{tr}[-2(\lambda L_{\mu }^{\left(1\right)}+(1-\lambda )L_{\mu }^{\left(2\right)})\\ +2W\Lambda W^{T}(\lambda L_{\mu }^{\left(1\right)}+(1-\lambda )L_{\mu }^{\left(2\right)})\\ +{\left(\lambda L_{\mu }^{\left(1\right)}+(1-\lambda )L_{\mu }^{\left(2\right)}\right)}^{2}] \end{array}$$
  The optimization problem can be rewritten as a standard quadratic programming formulation, which can be effectively solved with MATLAB *quadprog*.
Algorithm 1 presents the process of optimization for better understanding of scHoML.

Algorithm 1 High-order Laplacian matrix optimization for single-cell multi-omics data: scHoML

**Input**: Datasets:$\left\{X_{1},X_{2},\ldots ,X_{V}\right\}$, dimensionality of common embedding *c*, number of nearest neighbors *k*.

**Output**: Low-dimensional embedding H

1: Compute $L_{p}^{\left(i\right)}$ of each modal *X_p_*, p=1,2,…,V; i=1,2.

2: Initialize λ,W,Λ,μ.

3: **repeat**

4:   Update *λ* by solving optimization problem in Equation (6).

5:   Update W by solving Equation (7).

6:   Update Λ by solving Equation (8).

7:   Update H by solving Equation (9).

8:   Update μ by solving Equation (10).

9: **until** converge.

#### 3.1.2 Convergence and complexity

- **Convergence analysis**
  Since Laplacian matrix is a positive-semi-definite matrix, we can conclude that the objective function of scHoML takes zero as lower bound. Obtaining its global optimal solution is difficult, because the objective function is nonconvex. If alternative optimization framework is applied, the objective function value decreases while updating variables. Therefore, the algorithm will eventually converge to a local solution.
- **Complexity**
  The computational complexity of scHoML is mainly caused by SVD decomposition when updating *W* and *H*, and its corresponding complexity is $O(n^{3})$. Meanwhile, the complexity of updating *λ* and Λ is *O*(1) and *O*(*n*), respectively. Furthermore, to update *μ*, we need to solve a standard quadratic programming problem. Let *ε* be the precision of the result and *V* be the number of modals, the complexity of solving the quadratic programming problem is $O(\varepsilon^{-1}V)$. If the algorithm has been run for *t* iterations, the total complexity of our method is $O(t(n^{3}+n+\varepsilon^{-1}V))$. If $\varepsilon^{-1}\ll n^{2}$, the complexity of scHoML can be considered as $O(tn^{3})$.

### 3.2 Clustering with inferred low-dimensional representation

In the optimization of high-order neighborhood Laplace matrix, we simultaneously obtain a common low-dimensional embedding $H\in R^{n\times c}$ for the single-cell multi-modal data. The cell subpopulations can be identified from the matrix *H* through appropriate evaluation on the cellular relationships between cells.

Assume $H={\left[h_{1},h_{2},\ldots ,h_{n}\right]}^{T}\in R^{n\times c}$, we model the distance between cell *s* and cell *t* (s,t=1,2,…,n) as where ${\bar{h}}_{s}=\frac{1}{c}\sum_{j=1}^{c}x_{sj},\;s=1,2,\ldots ,n.$ and


$$\text{Dis}(s,t)=1-\frac{(h_{s}-{\bar{h}}_{s}){\left(h_{t}-{\bar{h}}_{t}\right)}^{T}}{\sqrt{(h_{s}-{\bar{h}}_{s}){\left(h_{s}-{\bar{h}}_{s}\right)}^{T}}\sqrt{(h_{t}-{\bar{h}}_{t}){\left(h_{t}-{\bar{h}}_{t}\right)}^{T}}}$$

$$\text{Dis}(s,t)=\sum_{k=1}^{c}|h_{s}(k)-h_{t}(k)|.$$

Agglomerative hierarchical clustering was performed on the constructed distance matrix to entangle the heterogeneity embedded in the cells.

An appropriate evaluation of the cluster number is critical. We here provide a grain to coarse design of the optimal cluster number. The cluster number is determined through solving the following optimization problems.

If the involved number of samples is small, we strive to evaluate the sample-specific Silhouette coefficient to measure the clustering matching degree and optimize the mean Silhouette coefficient of all samples to determine the best cluster number $c_{no}^{⋆}$: where $a_{k}(i)=\frac{1}{|C_{I}|-1}\sum_{j\in C_{I},i\neq j}d(i,j)$ is the average distance from the *i*th point to the other points in the same cluster *I* as *i*, and $b_{k}(i)={\text{min}}_{J\neq I}\frac{1}{\left|C_{J}\right|}\sum_{j\in C_{J}}d(i,j)$ is the minimum average distance from the *i*th point to points in a different cluster *J*, minimized over clusters. For different cluster number k∈K, we run agglomerative hierarchical clustering to generate different clustering results. If most samples have a high Silhouette value, the clustering solution is believed appropriate.


$$c_{no}^{⋆}={\text{argmax}}_{k\in K}\sum_{i=1}^{n}(b_{k}(i)-a_{k}(i))/\text{max}(a_{k}(i),b_{k}(i))$$

If the involved number of samples is relatively large, we propose statistical measures in terms of variance for evaluation of cluster number appropriateness. where $C_{q}$ is the set of all data in class *q*, $c_{q}$ is the central point of class *q*, $c_{E}$ is the central point of all data involved, and $n_{q}$ is the total number of data points in class *q*. It is reasonable that we evaluate inter-class variance and intra-class variance to determine the optimal cluster number $c_{no}^{⋆}$ when $\frac{\text{tr}(\sum_{q=1}^{k}n_{q}(c_{q}-c_{E}){\left(c_{q}-c_{E}\right)}^{T})/(k-1)}{\text{tr}(\sum_{q=1}^{k}\sum_{x\in C_{q}}^{}\left(x-c_{q}\right){\left(x-c_{q}\right)}^{T})/(n-k)}$ achieves maximum.


$$c_{no}^{⋆}={\text{argmax}}_{k\in K}\frac{\text{tr}(\sum_{q=1}^{k}n_{q}(c_{q}-c_{E}){\left(c_{q}-c_{E}\right)}^{T})/(k-1)}{\text{tr}(\sum_{q=1}^{k}\sum_{x\in C_{q}}^{}\left(x-c_{q}\right){\left(x-c_{q}\right)}^{T})/(n-k)},$$

## 4 Experiments

### 4.1 Datasets

We introduce a number of multi-modal single-cell data to test the performance of our proposed method.

- Simulation dataset 1.
  The dataset is obtained from Jin (2020), which consists of two simulated modals (paired scRNA-seq and scATAC-seq) at different noise levels. The ground truth data matrices were $X_{1}=W_{1}H,X_{2}=W_{2}H$, where $W_{1}=W_{1}+\rho E,W_{2}=W_{2}+\rho E$, *E* is the Gaussian noise with ρ=0.5.
  (11) $$W_{1}(i,j)=\{\begin{array}{cc} 1, & 1\leq i\leq 100,\;j=1;\\ & 151\leq i\leq 300,\;j=2;\\ & 501\leq i\leq 800,\;j=3;\\ 0, & \text{otherwise}; \end{array}$$
  (12) $$W_{2}(i,j)=\{\begin{array}{cc} 1, & 1\leq i\leq 500,\;j=1;\\ & 1001\leq i\leq 1500,\;j=2;\\ & 3001\leq i\leq 3800,\;j=3;\\ 0, & \text{otherwise}; \end{array}$$
  (13) $$H(i,j)=\{\begin{array}{cc} 1, & i=1,\;1\leq j\leq 70;\\ & i=2,\;71\leq j\leq 130;\\ & i=3,\;131\leq j\leq 200;\\ 0, & \text{otherwise}; \end{array}$$
  Dropouts were generated by a Bernoulli distribution on *X*_1_ and *X*_2_ with the probabilities $p_{1i},\;p_{2j}$, which were defined as $p_{1i}=e^{-\lambda_{1}x_{i}^{2}},p_{2j}=e^{-\lambda_{2}y_{j}^{2}}$, where *x_i_* is the mean expression level of the *i*th cluster of *X*_1_, *y_j_* is the mean expression level of the *j*th cluster of *X*_2_. Next, Gaussian noises were added to *X*_1_ and *X*_2_ as $X_{1}=X_{1}+\rho_{1}E,X_{2}=X_{2}+\rho_{2}E$, where noise parameter *ρ*_1_ in modal 1 varied from 3 to 5 with an increment 0.5, and noise parameter *ρ*_2_ varied from 0.2 to 1 with an increment 0.2. In total, there are 200 simulated cells, the number of attributes in modal 1 and 2 are 5000 and 2000, respectively. The cell-type number is 3.
- Simulation dataset 2.
  The dataset is obtained from Jin (2020), which consists of two simulated modals (paired scRNA-seq and scATAC-seq) where some clusters that were defined from epigenetic profile do not reflect transcriptomic distinctions. The ground truth data matrices were $X_{1}=W_{1}H,X_{2}=W_{2}H$, where $W_{1}=W_{1}+\rho E,W_{2}=W_{2}+\rho E$, *E* is the Gaussian noise with $\rho =0.5,\;x_{j}(n)=(j-1)(n-\mathrm{coph})$.
  (14) $$W_{1}(i,j)=\{\begin{array}{cc} 1, & 1+x_{j}(200)\leq i\leq 200+x_{j}(200);\\ 0, & \text{otherwise}; \end{array}$$
  (15) $$W_{2}(i,j)=\{\begin{array}{cc} 1, & 1+x_{j}(500)\leq i\leq 500+x_{j}(500);\\ 0, & \text{otherwise}; \end{array}$$
  The rank of *W*_1_ was set to be 3 and the rank of *W*_2_ (denoted as *K*_2_) varied from 3 to 7.
  (16) $$H(i,j)=\{\begin{array}{cc} 1, & 1+x_{j}(c)\leq i\leq x_{j}(c),\;j\leq K_{2}-1\\ & \text{or}\;1+x_{j}(c)\leq i\leq n,\;j=K_{2};\\ 0, & \text{otherwise}; \end{array}$$
  Similar to the above-mentioned procedures, dropouts on both *X*_1_ and *X*_2_ were generated with $\lambda_{1}=0.05,\lambda_{2}=0.025$ and added Gaussian noise with $\rho_{1}=2,\rho_{2}=1$. In addition, if the values in *X*_2_ with dropouts were greater than 0.7, we set the values to be 1, otherwise 0. In total, there are 500 simulated cells with four different cell types, the number of attributes in modal 1 and 2 are 5000 and 2000, respectively.
- Mouse embryonic stem cells (mESCs) data.
  The dataset was obtained from 77 mESCs, including 13 cells cultured in “2i” media and 64 serum-grown cells, which were profiled by parallel scM&T-seq technique (Angermueller 2016).
- pbmc_inhouse data.
  It is a CITE-seq dataset extracted from a healthy donor under IRB approval from the University of Pittsburgh (Wang *et al.* 2020). We follow the instructions of cell type identification using well-defined markers, and removed those cells with uncertain cell types. In the dataset, there are 1242 cells in total, containing five different cell types.
- pbmc_10X data.
  The multi-modal single-cell data (CITE-seq dataset) was downloaded from 10× Genomics website. A total of 7865 human peripheral blood mononuclear cells (PBMCs) with 14 surface protein markers are included in the dataset in addition to matched scRNA-seq data. Cells with uncertain cell types were removed. In total, there are 6661 cells involved, containing seven different cell types: B cells, CD14+ monocytes, CD16+ monocytes, CD4+ T cells, CD8+ T cells, dendritic cells, and natural killer (NK) cells.

### 4.2 Methods for comparison

We compare our proposed method scMoHL with state-of-the-art method scAI (Jin 2020), for deconvoluting cellular heterogeneity from parallel transcriptomic and epigenomic profiles. Apart from that, we introduce a number of methods for dealing with multi-modal data for comparison. The methods for comparison are listed as follows:

- SCbest: a single-view spectral clustering applied for all the views with the best clustering for output.
- MSE (Xia *et al.* 2010): a multi-view spectral embedding method. We set the parameter *r* in the range of [2,3,…,10] and report the best result.
- CoregSC (Kumar and Daumé 2011): a multi-view spectral clustering method based on co-training strategy. We set the parameter λ=0.01 for clustering.
- AASC (Huang *et al.* 2012): a multi-view spectral clustering method for optimizing linear combination of affinity matrices.
- RMSC (Xia *et al.* 2014): a multi-view spectral clustering combined with Markov chain. The parameter *λ* is set as 0.005 for clustering.
- AMGL (Nie *et al.* 2016): a auto-weighted multiple graph learning method for multiple views.
- AWP (Nie *et al.* 2018): a multi-view spectral clustering based on spectral rotation technique.
- scAI (Jin 2020): a regularized matrix-factorization framework for single-cell multi-omics data integration.
- OPMC (Liu *et al.* 2021): a multi-view matrix factorization clustering.

### 4.3 Computational results

For performance evaluation, we introduced two popular measures: Adjusted Rand Index (ARI) and Normalized Mutual Information (NMI) for comparing the clustering accuracy. Table 1 reports the ARI measures for the compared methods applying in the considered datasets. Table 2 reports the NMI measures for the compared methods applying in the considered datasets. In the construction of scMoHL, there are two parameters involved: the scale parameter *σ* and the parameter *k* in KNN graph. We set the scale parameter *σ* as the sample standard deviation of dataset, and the parameter *k* required by the KNN matrix is set to 10 for small datasets and 100 when sample size is relatively large. Modal-specific Laplacian matrices are computed on the preprocessed datasets through standard normalization and dimension reduction with principal component analysis. As for the multi-modal integration methods, SCbest, MSE, CoregSC, AASC, RMSC, AMGL, and *k*-means clustering methods are applied on the integrated embedding matrix obtained by these methods, hence we report the averaged ARI and NMI values running 20 times for performance comparison. And the averaged ARI and NMI values with standard deviations are reported. AWP can ensure a stable clustering result, hence the standard deviation is 0. In scAI method, a low-dimensional representation matrix *H* for the multi-omics single-cell data was obtained through regularized matrix factorization framework. The heterogeneity of cells is then identified by clustering through the low-dimensional representation matrix *H* using the Leiden community detection method, with default resolution parameter setting of 1. The resolution parameter has a great effect on the cluster number evaluation for the dataset. When we set the default resolution parameter 1 in experiments, most clustering results turned to be single cluster instead, distorting the original heterogeneity in datasets. Hence we use resolution parameter 0.1 in Leiden algorithm (scAI-Leiden) on the best *H* chosen (scAI is applied to each dataset 10 times with different seeds) in the comparisons. Besides, we also introduce consensus hierarchical clustering method on the matrix *H* obtained by scAI (scAI-hc). Regarding the cluster number, if the cluster number is a parameter as input including scAI-hc, we use the true cluster number for comparison. In simulation_data1, the true cluster number is 3, and the estimated cluster number by Leiden algorithm and scHoML are both 3. For simulation_data2, where the true cluster number is 4, the estimated cluster number by scHoML is 4. But for Leiden algorithm, the estimated cluster number is 5. mESC dataset has two different cell states, but in Leiden algorithm, the estimated cluster number is equal to the number of cells in the dataset, namely, 77. scHoML can accurately estimate the cluster number. For pbmc_inhouse and pbmc_10X data, where the true cluster numbers are 5 and 7, respectively, scAI-Leiden estimated the cluster number to be 13 and 77 instead. Overall, scAI-Leiden tends to overestimate the heterogeneous groups inside datasets.

**Table 1.** Performance comparisons of different methods in terms of ARI.

		Dataset			
Algorithm	Simulation data1	Simulation data2	mESC	pbmc_10X	pbmc_inhouse
SCbest	0.2426 ± 0.0233	0.2323 ± 0.0072	0.3764 ± 0.0000	0.7214 ± 0.0411	0.6679 ± 0.0742
MSE	0.2709 ± 0.0050	0.0111 ± 0.0059	0.3764 ± 0.0000	0.7161 ± 0.0133	0.6807 ± 0.0502
CoregSC	0.2529 ± 0.0080	0.1368 ± 0.0280	0.1716 ± 0.0628	0.7162 ± 0.0164	0.5152 ± 0.1351
AASC	0.1755 ± 0.0044	0.0063 ± 0.0089	0.7022 ± 0.0235	0.6805 ± 0.0799	0.6027 ± 0.0290
RMSC	0.0423 ± 0.0094	0.0745 ± 0.0050	0.3468 ± 0.0000	0.5724 ± 0.0001	0.5212 ± 0.0266
AMGL	0.1424 ± 0.0892	−0.0246 ± 0.0123	0.4869 ± 0.4691	0.6332 ± 0.1047	0.7275 ± 0.1452
AWP	0.3338 ± 0.0000	0.1154 ± 0.0000	0.2416 ± 0.0000	0.7230 ± 0.0000	0.5693 ± 0.0000
OPMC	0.0241 ± 0.0463	0.6927 ± 0.3311	0.2269 ± 0.2012	0.6133 ± 0.0648	0.8778 ± 0.1095
scAI-hc	0.9391 ± 0.0000	0.6587 ± 0.0000	0.5480 ± 0.0000	0.5131 ± 0.0000	0.7875 ± 0.0000
scAI-Leiden	0.9539 ± 0.0000	0.8042 ± 0.0000	0 ± 0.0000	0.0738 ± 0.0000	0.3317 ± 0.0000
scHoML	0.9854 ± 0.0000	1 ± 0.0000	0.9350 ± 0.0000	0.8737 ± 0.0000	0.9523 ± 0.0000

**Table 2.** Performance comparisons of different methods in terms of NMI.

		Dataset			
Algorithm	Simulation data1	Simulation data2	mESC	pbmc_10X	pbmc_inhouse
SCbest	0.2630 ± 0.0149	0.2572 ± 0.0047	0.3894 ± 0.0000	0.8144 ± 0.0070	0.8456 ± 0.0470
MSE	0.3196 ± 0.0098	0.0404 ± 0.0108	0.3894 ± 0.0000	0.8149 ± 0.0004	0.8536 ± 0.0325
CoregSC	0.2796 ± 0.0026	0.1650 ± 0.0295	0.2742 ± 0.0341	0.8125 ± 0.0057	0.6456 ± 0.1145
AASC	0.2456 ± 0.0027	0.0592 ± 0.0120	0.6198 ± 0.0200	0.7374 ± 0.0398	0.6877 ± 0.0195
RMSC	0.1130 ± 0.0295	0.0688 ± 0.0027	0.3719 ± 0.0000	0.6705 ± 0.0000	0.6179 ± 0.0186
AMGL	0.2313 ± 0.0787	0.1140 ± 0.0231	0.5166 ± 0.4328	0.7106 ± 0.0574	0.8615 ± 0.0701
AWP	0.3704 ± 0.0000	0.1703 ± 0.0000	0.3122 ± 0.0000	0.8078 ± 0.0000	0.7225 ± 0.0000
OPMC	0.0329 ± 0.0450	0.7343 ± 0.3040	0.2372 ± 0.1989	0.6939 ± 0.0149	0.8815 ± 0.0298
scAI-hc	0.9112 ± 0.0000	0.7892 ± 0.0000	0.5003 ± 0.0000	0.6232 ± 0.0000	0.7906 ± 0.0000
scAI-Leiden	0.9348 ± 0.0000	0.8080 ± 0.0000	0.3233 ± 0.0000	0.5128 ± 0.0000	0.7170 ± 0.0000
scHoML	0.9764 ± 0.0000	1 ± 0.0000	0.8729 ± 0.0000	0.8374 ± 0.0000	0.9470 ± 0.0000

#### 4.3.1 Overall performance comparison

Aside from scMoHL, all the compared algorithms demonstrate specific patterns, with no dominant methods.

scAI demonstrates obvious superiority compared to SCbest, MSE, CoregSC, AASC, RMSC, AMGL, AWP, and OPMC for simulated datasets. But the performance of scAI is not satisfactory when applied in mESC and pbmc datasets. SCbest as a single-view clustering method performs in an unstable way, where the ARI values obtained for the two simulated datasets and mESC dataset are unsatisfactory, revealing the complex structure of multi-modals in the respected datasets. But it is interesting to see that on pbmc data, SCbest outperforms most of the compared methods including scAI, indicating that in this dataset, there exists some modal showing a clear relationship among data. MSE as a multi-modal integration method performs similarly as SCbest. In particular for simulation data2 when the single-view clustering result showing 0.2323 in ARI value on average, MSE cannot learn a better integration of modals, getting only 0.0111 in averaged ARI value. For AASC, the clustering result on mESC data is the best excluding scHoML, showing 0.7022 in averaged ARI value. The performance of RMSC and AMGL resembled with each other on simulated datasets. When applied on real-world datasets, RMSC and AGML cannot compete with scAI in ARI values on mESC data. RMSC and AGML show better performance than scAI in ARI values and NMI values on pbmc data. For AWP algorithm, the best clustering performance is achieved on pbmc_10X dataset, 0.7230 in ARI value. OPMC algorithm shows the best clustering performance in pbmc_inhouse data, while has poor discrimination power in other datasets.

When we compare single-view clustering-based algorithm (SCbest) with the other multi-modal clustering methods, some conclusions can be made as follows. First, different modal may reveal the data in different perspectives, there are cases when some particular modal shows a clear relationship among data. Second, integration methods may not fully integrate the proper information embedded in modals, showing unsatisfactory result compared to single-view based clustering method. Taking CoregSC for example, the results in mESC data, pmbc_inhouse and pbmc_10X data are inferior to that of SCbest. Third, appropriate evaluation of the data relationship is of critical significance for entangling the heterogeneity described by multi-modals. Among all the compared methods, scHoML as a graph-based embedding method provides a better description on the relationship between cells, showing that the incorporation of high-order correlation contributes in a positive manner for relationship description.

#### 4.3.2 Comparison with scAI: aggregation and integration method for parallel single-cell multi-omics data

scAI demonstrates explicit superiority compared to other traditional multi-view data clustering methods for simulation datasets. For simulation datasets, scAI ranks the second best, slightly inferior to scHoML. In real-world datasets such as mESC data, scAI with consensus hierarchical clustering method ranks the third in clustering accuracy in terms of ARI and NMI values. It is interesting to see that in pbmc-inhouse data and pbmc-10X data, scAI is not satisfactory. The computational efficiency is also restricted in scAI when the datasets contain large population of cells. When we compare different clustering methods in conjunction with scAI, we have the following findings. scAI with consensus hierarchical clustering (scAI-hc) outperforms scAI with Leiden clustering (scAI-Leiden) in cellular population heterogeneity analysis in an overall manner. For simulation datasets, scAI-Leiden perform in a similar way with scAI-hc. While for real-world datasets, scAI-Leiden perform in a unsatisfactory way. One possible explanation may be that the resolution parameter play an important role in the performance of scAI-Leiden method, especially in tuning the number of clusters to be detected. In the experiments with resolution parameter 0.1, the tuned number of clusters is more reasonable compared to default resolution parameter 1 for many datasets. Hence we used resolution parameter 0.1 for performance illustration. Second, we further checked the influence of resolution parameter on the clustering performance for datasets as shown in Supplementary Table S3. We found that default resolution parameter 1 would be appropriate in traditional cases, but may also fail in many new cases. In our experiments with the considered datasets, they all perform unsatisfactory results. Besides, different datasets have different optimal resolution parameters. The estimation and determination of optimal resolution parameter would become an interesting problem.

In the comparison, scHoML shows the best performance for integration in both simulation datasets and real-world single-cell multi-omics data when the performance is evaluated in ARI and NMI measures. As a nonlinear relationship modeling framework, scHoML used Laplacian matrix to model the relationship in multi-modal single-cell data. In particular, the incorporation of high-order neighborhood Laplacian matrix in optimization contributes to a better description of the geometric structure of the complex multi-modal data. Besides, scHoML can robustly represent the noisy, sparse multi-omics data in a unified low-dimensional embedding space. The cluster number determination strategy with sample-specific Silhouette coefficient for small sample problems as well as variance-based statistical measure offers a flexible way for accurately estimating the intrinsic clusters in the data. However, the computational complexity would become an unavoidable issue if the involved number of cells is large, because the time complexity is proportional to the number of cells *n*, which is $O(n^{3})$.

#### 4.3.3 Common embedding performance evaluation

All the compared methods attempt to find a common low-dimensional embedding for the single-cell multi-omics data, hence we compared 2D visualization of aggregated low-dimensional embeddings by different methods to evaluate the embedding capabilities of the considered methods. All the figures are attached in Supplementary Files.

**Figure 1.**
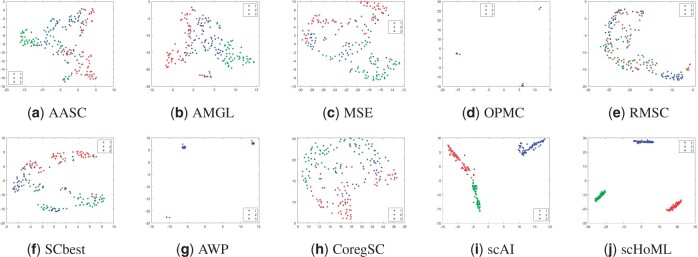
tSNE plots of different methods in obtaining low-dimensional embeddings for simulation data1. Subfigures (a) to (j) represent the different methods indicated by the subtitles.

**Figure 2.**
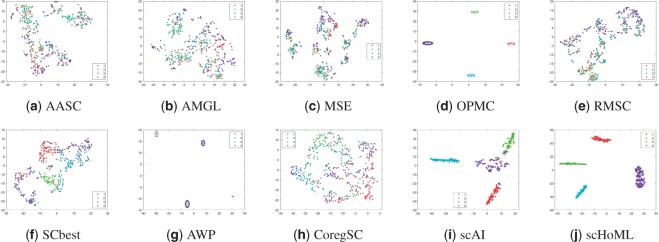
tSNE plots of different methods in obtaining low-dimensional embeddings for simulation data2. Subfigures (a) to (j) represent the different methods indicated by the subtitles.

**Figure 3.**
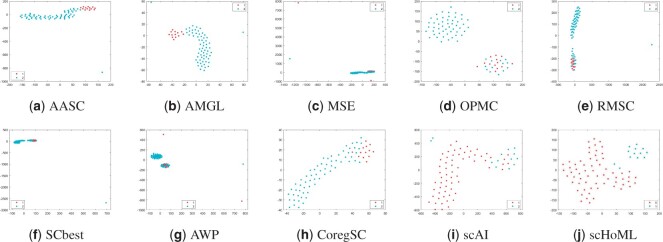
tSNE plots of different methods in obtaining low-dimensional embeddings for mESC data. Subfigures (a) to (j) represent the different methods indicated by the subtitles.

**Figure 4.**
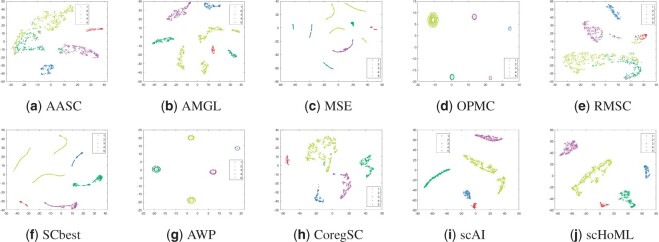
tSNE plots of different methods in obtaining low-dimensional embeddings for pbmc_inhouse data. Subfigures (a) to (j) represent the different methods indicated by the subtitles.

**Figure 5.**
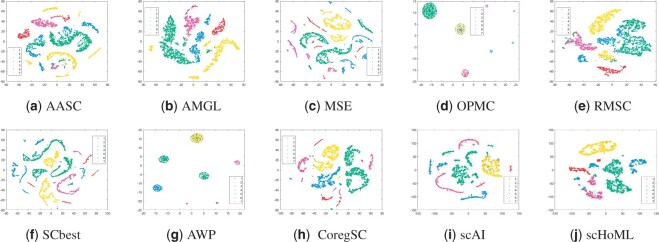
tSNE plots of different methods in obtaining low-dimensional embeddings for pbmc_10X data. Subfigures (a) to (j) represent the different methods indicated by the subtitles.

Figures 1–5 show the visualization of aggregated low-dimensional embeddings by different methods using tSNE. Different color represents different true clusters in the dataset. Figures 1 and 2 refer to the tSNE plots for simulation datasets with considered methods. Traditional multi-view clustering methods, such as SCbest, MSE, CoregSC, AASC, RMSC, AMGL, AWP, and OPMC failed to decipher the heterogeneity in the cells, where the cell subpopulations were indistinguishable in the recovered low-dimensional space by those methods. However, scAI can obtain a proper low-dimensional embedding matrix *H* showing appropriate relationship in the cells, where the cells are almost distinguishable. scHoML demonstrates clear superiority in getting common embedding information for the two simulation datasets, and the cell subpopulations were clearly distinguishable in the low-dimensional space when using the aggregated data.

Figure 3 shows tSNE plot of different methods in obtaining low-dimensional embeddings for mESC data. AASC and AMGL perform similarly, and the cells tend to show distinguishable properties. scHoML clearly help recover a satisfactory low-dimensional embedding for mESC data generated by parallel scM&T-seq technique. Other methods including scAI cannot guarantee a proper embedding for mESC data, where different types of cells tend to mix with each other.

As shown in Fig. 4, for pbmc_inhouse data, AMGL, SCbest, and MSE perform quite similarly and the tSNE plots for the three methods share similar pattern formations, where most of the cells are distinguishable. scHoML undoubtedly demonstrates superiority compared to the remaining nine methods in aggregated low-dimensional representation for the 1242 pbmc cells profiled by CITE-seq.

Figure 5 corresponds to the aggregated low-dimensional embedding for PBMC_10X data, where the multi-modal single-cell data (CITE-seq dataset) was downloaded from 10× Genomics website containing 6661 human PBMCs cells. When the number of cells increase, the data become more complicated and the cells are more heterogeneous. Methods include OPMC and RMSC, AWP tend to mix the cells. Apart from scHoML, CoregSC shows the best aggregation performance where the same type of cells are more compactly scattered though for some particular types, the cells are diversely scattered.

**Figure 6.**
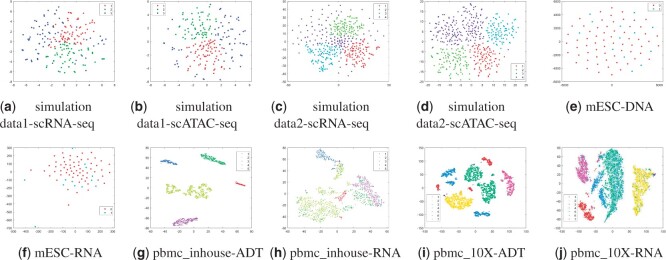
tSNE plots for original multi-omics data. Subfigures (a) to (j) represent the different methods indicated by the subtitles.

Take a further look at the intrinsic complexity of the multi-omics data, we analyze the tSNE plots for original data in all considered modals. Due to the inherent sparsity and noise in the data, the cells were not well separated in the scRNA-seq data and the scATAC-seq data using t-SNE, for simulation datasets as shown in subfigs (a) and (b) for simulation data1 and (c) and (d) for simulation data2 in Fig. 6. Also, for mESC data, the cell populations are mixed, as shown in subfigs (e) and (f). However, the aggregated low-dimensional data generated by scHoML help capture heterogeneity between different types of cells, as shown in subfig (j) in Figs 1–3. For pbmc_inhouse data, the modal described by ADT features can clearly differentiate cell types, while the modal described by RNA is quite noisy and makes the data analysis complicated. It is interesting to see that for scAI and many of the traditional multi-view data integration methods, such as MSE, AASC, RMSC, and OPMC, the aggregated matrix *H* obtained by those methods failed to give play to the descriptive advantages of ADT features, but quite influenced by the noisy RNA data. scHoML tends to obtain the satisfactory common embedding for pbmc_inhouse data. Similar results can be discovered by pbmc_10X data.

## 5 Discussions

From the computational results, we can confirm the robustness and effectiveness of scHoML in dealing with single-cell multi-omics data under different signal-to-noise ratio scenarios. In the following, we discuss the feasibility and effectiveness of multi-modal and high-order Laplacian matrix optimization in scHoML. While only considering one mode of single-cell data, we have the following observations. Different datasets demonstrate different characteristics. For simulation data1 (subfigs a and b), simulation data2 (subfigs c and d), and mESC data (subfigs e and f) as shown in Fig. 6, both modals are noisy. scHoML, however, can overcome the influence of noise effect, robustly integrate the noisy modals to generate a clear common embedding in low dimensions as shown in Figs 1–3 subfig (j). Slightly inferior to scHoML, scAI can similarly recover the geometric distribution of cells after data integration, shown in Figs 1–3 subfig (i). The multi-view clustering-based algorithms seem to be considerably influenced by the noisy data, and the performance of integration sometimes cannot compete with single-mode clustering method SCbest. For pbmc_inhouse data or pbmc_10X data, the ADT modal is relatively clear for cell-type differentiation on tSNE shown in Fig. 6 subfigs (g) and (i). It is shown in Figs 4 and 5 that multi-view clustering-based algorithms, such as AWP and CoregSC can learn the clear relationship revealed by the ADT modal, demonstrating a relatively acceptable performance. However, scAI seems to be influenced by the clustering algorithm, in particular for Leiden algorithm. When the resolution parameter differs, the performance fluctuates with large variance. scHoML among all the compared partners shows the stable and robust performance. We conclude that when modals are noisy, scHoML can dig inside the intrinsic geometric relationship and learn a clear common embedding; when multi-modal data contains clear modal information, scHoML has the ability of not being influenced by noisy modals. When only considering the first-order Laplacian matrix optimization, we can check the performance of MSE whose optimization objective is ${\text{argmin}}_{H,a}\sum_{p=1}^{V}a_{p}^{r}\text{tr}(H^{T}L_{p}H)$. From the tSNE plots in Figs  1–5 as well as Tables 1 and 2, we came to know that when all the involved modals are noisy, MSE did not have the ability to extract the intrinsic relationship of cells accurately. However, when incorporating high-order Laplacian information, we model the hidden high-order connection information among data in scHoML, and the learned low-dimensional data provide a better representation of the original noisy modals.

**Figure 7.**
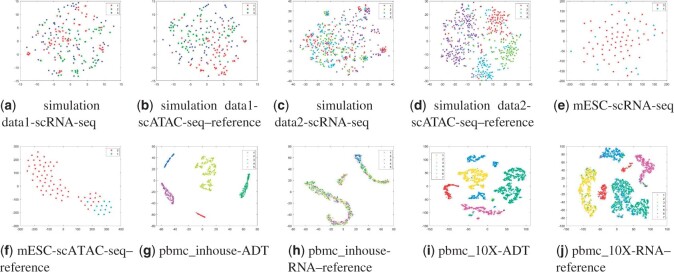
tSNE plots for embedding data by UnionCom: Case 1. Subfigures (a) to (j) represent the different methods indicated by the subtitles.

**Figure 8.**
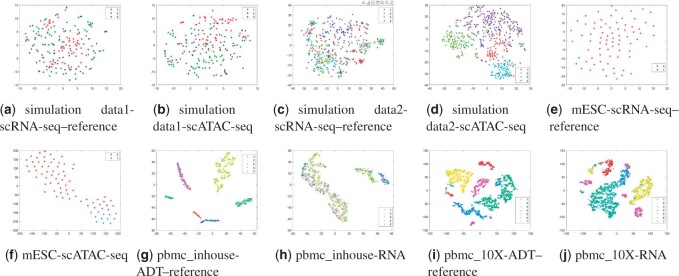
tSNE plots for embedding data by UnionCom: Case 2. Subfigures (a) to (j) represent the different methods indicated by the subtitles.

Besides, we compared scHoML to a robust single-cell muti-omics integration method UnionCom (Cao *et al.* 2020) in terms of embedding capability. In UnionCom, a reference modal within the dataset should be given in advance for further processing. We therefore have included all the cases when each modal is regarded as reference modal in the comparison. It can be shown in Figs 7 and 8 that the projected low-dimensional modals cannot compete with scHoML in tSNE showing. When all the modals are noisy, the common low-dimensional space learned by UnionCom is also noisy. However, if some modals show high data quality in the multi-omics data, UnionCom can guarantee a relative clear representation of the multi-omics data.

- **Heterogeneity analysis**
  We further hope to dissect the heterogeneity analysis results provided by scHoML. It is interesting to see that, for pbmc_10X data, the original data contains 1112 CD8+ T cells as a single cluster. However, scHoML divides the 1112 cells into two major clusters, with one cluster containing 766 cells, the other cluster containing 322 cells. Hence, we conduct statistical analysis to compare the differences between the two subclusters. In the ADT-based data, we did one-sided two-sample Kolmogorov–Smirnov goodness-of-fit hypothesis test on the two populations generated by scHoML, and select the representative markers which rejects the null hypothesis that F1(x)=F2(x) as the corresponding true (but unknown) population CDFs at the 5% significance level. The representative markers in subcluster 1 (766 cells) include “TIGIT,” “CD3+,” “CD4+,” and “CD8+,” which are reported marker genes for annotating exhausted CD8+ T cells (Deng *et al.* 2021). A further analysis on the representative genes for cluster one through one-sided two-sample Kolmogorov–Smirnov goodness-of-fit hypothesis test leads to a filtration of top-ranked genes “CCL5,” “HLA-DRB1,” “GZMH,” “HLA-DPA1,” and “NKG7.” It is well-established (Ren *et al.* 2021) that for CD8+ T cells, the major pTRTs were exhausted T cells and exhibited high heterogeneity. And in our dataset, we successfully identify the subcluster that is consistent with cluster C7 harboring a low frequency of terminal Tex cells and high frequency of “CD8+ZNF683+CXCR6+” Trm cells, dominated by naive T cells. It demonstrates the capability of scHoML in identifying the heterogeneity pattern embedded in the noisy single-cell multi-omics data.
- **Cellular state identification**
  We conduct statistical analysis to compare the differences between specific cluster and the remaining clusters by scHoML, to investigate the potential of scHoML in cellular state identification. We performed *t*-test of the hypothesis that the two independent samples generated by the specific cluster and the remaining clusters come from distributions with equal means, and returns the result of the test in *H*. Here *H *=* *0 indicates that the null hypothesis (“means are equal”) cannot be rejected at the 5% significance level. Here *H *=* *1 indicates that the null hypothesis can be rejected at the 5% level. To be specific, when we consider hypothesis test on cluster 3 and the remaining clusters, in the ADT data with 14 attributes, we filter the differentially expressed attributes for cluster 3 through hypothesis testing. They are “CD16,” “CD56,” “CD45RA,” etc. It is well verified that “CD16” and “CD56” are classical markers for NK cells (Dege *et al.* 2020; Harper 2021). A further analysis on the representative genes for cluster 3 through *t*-test leads to a filtration of top-ranked genes “FCGR3A,” “GNLY,” “SPON2,” “FGFBP2,” and “TKTL1.” “FCGR3A,” according to UniProtKB/Swiss-Prot, mediates IgG effector functions on NK cells (Lee *et al.* 2015). The protein product of “GNLY” is present in cytotoxic granules of cytotoxic T lymphocytes and NK cells, revealing the relationship between “GNLY: and NK cells. These findings indicate the capability of scHoML in extracting key molecules for cellular-type/state identification. Besides, we further conducted analysis on the downstream feature selection capability for the compared methods. The comparison methods include OPMC, AWP, and scAI, because the clustering results are relatively stable and robust, which are more suitable for stable feature selection. Similarly, we performed *t*-test on cluster i,i=1,2,… and the remaining clusters, in the ADT data with 14 attributes, to annotate the cluster-specific markers by the compared methods. In OPMC method, the markers identified using “*t*-test” for clusters 1–7 are as follows: cluster 1: “CD8a,” “CD45,” “CD3,” “CD127,” “TIGIT”; cluster 2: “CD8a,” “CD45,” “TIGIT,” “PD-1,” “CD3”; cluster 3: “CD19,” “CD45,” “CD56,” “CD16,” “TIGIT”; cluster 4: “CD3,” “CD4,” “CD127,” “CD45,” “CD25”; cluster 5: “CD14”; cluster 6: “CD56,” “CD45,” “CD16,” “TIGIT,” “CD19”; cluster 7: “CD8,” “CD127,” “CD45,” “CD3,” “PD-1.” We can see that in the identified markers, cluster 1 and cluster 2 have many common markers, indicating that OPMC cannot distinguish the cells in a clear manner where some clusters share similar patterns. In AWP method, the markers identified using “*t*-test” for clusters 1–7 are as follows: cluster 1: “CD8a,” “CD127,” “CD3,” “TIGIT,” “PD-1”; cluster 2: “CD4,” “CD45,” “PD-1,” “CD127”; cluster 3: “CD14”; cluster 4: “CD19,” “CD45,” “CD56,” “CD16,” “TIGIT”; cluster 5: “CD19,” “CD45”; cluster 6: “CD16,” “CD56,” “CD45,” “TIGIT,” “CD15”; cluster 7: “CD3,” “CD4,” “CD127,” “CD45,” “CD15.” We can see that the identified markers in cluster 5 are a subset of markers, meaning that AWP cannot distinguish the cells in a clear manner as well. In scAI-hc, the markers identified using “*t*-test” for clusters 1–7 are as follows: cluster 1: none; cluster 2: “CD4,” “CD3,” “CD45,” “CD14,” “CD25,” “CD127,” “PD-1”; cluster 3: “CD56,” “CD45”; cluster 4: “CD56,” “CD45,” “CD19,” “CD16,” “TIGIT,” “CD15”; cluster 5: “CD45”; cluster 6: “CD14”; cluster 7: “CD8a,” “CD127,” “CD3,” “TIGIT,” “PD-1,” “CD45,” “CD15.” We can see that the identified markers in cluster 5 are a subset of markers cluster 3, the identified markers in cluster 3 are a subset of markers, cluster 4, cluster 2 and cluster 7 have a lot of common markers, indicating that scAI-hc cannot help distinguish the cells in a clear manner as well. In scHoML method, the markers identified using “*t*-test” for clusters 1–7 are as follows: cluster 1: “CD19,” “CD45”; cluster 2: “CD8a,” “CD127,” “CD3,” “CD45”; cluster 3: “CD16,” “CD56,” “CD45,” “TIGIT,” “CD15”; cluster 4: “CD3,” “CD4,” “CD127,” “CD45,” “CD25”; cluster 5: “CD14”; cluster 6: “CD8a,” “CD45,” “TIGIT,” “PD-1,” “CD3”; cluster 7: “CD19.” Cluster 7 only contains 0.02% number of cells. We can see that the identified markers in each cluster have distinct characteristics, for example, “CD16,” “CD56,” “TIGIT” in cluster 3 are typical biomarkers for NK cells, demonstrating the ability of scHoML in identifying meaningful biomarkers.

## 6 Conclusions

In this study, we propose a multi-modal high-order neighborhood Laplacian matrix optimization framework for integrating the multi-omics single-cell data: scMoHL. scHoML can robustly model the complex data structures and represent the noisy, sparse multi-omics data in a unified low-dimensional embedding space. Experiments on simulated datasets as well as real single-cell multi-omics data reveal that scHoML faithfully aligned heterogeneous modalities. The embedded data can further be utilized for heterogeneity analysis as well as cellular state identification, expecting to shed light on intriguing studies for revealing significant mechanisms among cells.

## Supplementary Material

btad414_Supplementary_Data
