# Correction to: Robust joint clustering of multi-omics single-cell data via multi-modal high-order neighborhood Laplacian matrix optimization

**DOI:** 10.1093/bioinformatics/btad554

**Published:** 2023-09-12

This is a correction to: Hao Jiang and others, Robust joint clustering of multi-omics single-cell data via multi-modal high-order neighborhood Laplacian matrix optimization, *Bioinformatics*, Volume 39, Issue 7, July 2023, btad414, https://doi.org/10.1093/bioinformatics/btad414

The supplementary data file originally published with this manuscript has been updated to a new version, which includes an additional term in Equation (2) and Equation (7).

