# Supplementary material for: Robust joint clustering of multi-omics single-cell data via multi-modal high-order neighborhood Laplacian matrix optimization
Source: Bioinformatics. 2023 Jun 29;39(7):btad414. doi: 10.1093/bioinformatics/btad414 (PMC10329495; doi:10.1093/bioinformatics/btad414)
Supplement: btad414_Supplementary_Data [file btad414_supplementary_data.pdf]

Supplementary File

1 Methods and Optimization Details

We present the method for integrating multi-omics data, to optimize the fused Laplacian matrix, as well as obtaining the low-dimensional representation for the multi-omics data.

For single modal data, spectral clustering can be realized through solving optimization problem with given laplacian matrix L. In terms of multi-omics single cell data, the proposed Laplacian matrix $\mathcal{L}^*$ approximated by linear combination of different order modal-specific laplacian matrices in the form $\sum_{i=1}^{U} \lambda_i \mathcal{L}_\mu^{(i)}$ has several parameters yet to be determined.

How to automatically determine the parameters embedded in Laplacian matrix $\mathcal{L}^*$ and seek better representation capability of the common embedding for multi-modal data thus constitutes a critical challenge. Motivated by the framework of spectral clustering, we propose the optimization objective as minimization of $\mathrm{tr}(H^T \mathcal{L}^* H)$, while simultaneously seek optimized H as the low-dimensional embedding for the multi-modal data, and the optimization problem can be expressed as follows:

$$
\begin{aligned}
\min_{\boldsymbol{\lambda}, H, \boldsymbol{\mu}} \ & \mathrm{tr}(H^T \mathcal{L}^* H) + \|\mathcal{L}^* - \sum_{i=1}^{U} \lambda_i \mathcal{L}_\mu^{(i)}\|_F^2 \\
& s.t.\ \mathcal{L}_\mu^{(i)} = \sum_{p=1}^{V} \mu_p L_p^{(i)} (i = 1, 2, \cdots, U), \\
& \mathcal{L}^* (\text{positive-semi-definite}), \mathcal{L}_{jk}^* \leq 0, j \neq k \\
& H \in \mathbb{R}^{n \times c}, H^T H = I_c \\
& \boldsymbol{\mu} = [\mu_1, \mu_2, \ldots, \mu_V]^T, \|\boldsymbol{\mu}\|_1 = 1, \boldsymbol{\mu} \geq 0 \\
& \boldsymbol{\lambda} = [\lambda_1, \lambda_2, \ldots, \lambda_U]^T, \|\boldsymbol{\lambda}\|_1 = 1, \boldsymbol{\lambda} \geq 0.
\end{aligned}
\tag{1}
$$

1.1 Multi-Modal Laplacian Matrix Optimization

High-order Laplacian matrix can model the hidden high-order connection information among data, but the value of order needs properly selected as too high order may distort the original relationship embedded in the data set. Hence, we focus on integration of Laplacian matrix in first-order and second-order, to preserve global data structure in a better manner, as well as improving learning

performance. However, the positive-semi-definite property of $\mathcal{L}^*$ added in the constraints of the optimization problem makes the optimization problem hard and inefficient to solve. Taking into consideration on the original definition of Laplacian matrix $I_n - D^{-1/2}W_1D^{-1/2}$, and the symmetric property of W_1 that can be decomposed into eigen-matrix form $W_1 = \tilde{U}\Lambda\tilde{U}^T$, the optimization term $\mathcal{L}^*$ can be reformulated with $I_n - W\Lambda W^T$. We here present a generalized version of the optimization problem to improve the experimental performance:

$$\begin{aligned}
\min_{\lambda,W,\Lambda,H,\boldsymbol{\mu}} \quad & \mathrm{tr}(H^T(I_n - W\Lambda W^T)H) + \alpha\boldsymbol{\mu}^T M\boldsymbol{\mu} \\
& + \|I_n - W\Lambda W^T - (\lambda\mathcal{L}_\mu^{(1)} + (1-\lambda)\mathcal{L}_\mu^{(2)})\|_F^2 \\
& s.t.\ \mathcal{L}_\mu^{(i)} = \sum_{p=1}^{V}\mu_p L_p^{(i)} (i = 1, 2), \\
& W, H \in \mathbb{R}^{n\times c}, W^TW = I_c, H^TH = I_c \\
& \boldsymbol{\mu} = [\mu_1, \mu_2, \ldots, \mu_V]^T, \|\boldsymbol{\mu}\|_1 = 1, \boldsymbol{\mu} \geq 0 \\
& 0 \leq \Lambda_{kk} \leq 1, k = 1, 2, \ldots, c, 0 \leq \lambda \leq 1.
\end{aligned} \tag{2}$$

Here Λ is a diagonal matrix, and $0 \leq \Lambda_{kk} \leq 1$ makes sure the optimization stable.

Optimization Framework

Taking into consideration on the non-convexity of the above problem, we propose alternative optimization framework to solve the problem by updating each variable iteratively. For the convenience of optimization, we rewrite $\|I_n - W\Lambda W^T - (\lambda\mathcal{L}_\mu^{(1)} + (1-\lambda)\mathcal{L}_\mu^{(2)})\|_F^2$ into the following form:

$$\begin{aligned}
& \mathrm{tr}[I_n - 2W\Lambda W^T - 2(\lambda\mathcal{L}_\mu^{(1)} + (1-\lambda)\mathcal{L}_\mu^{(2)}) \\
& + 2W\Lambda W^T(\lambda\mathcal{L}_\mu^{(1)} + (1-\lambda)\mathcal{L}_\mu^{(2)}) \\
& + W\Lambda^2W^T + (\lambda\mathcal{L}_\mu^{(1)} + (1-\lambda)\mathcal{L}_\mu^{(2)})^2].
\end{aligned}$$

The optimization process consists of the following five steps.

- **Updating** λ: Fixing $W, \Lambda, H, \boldsymbol{\mu}$, the update of λ can be realized through solving the optimization problem:

$$\begin{aligned}
\min_{0\leq\lambda\leq1} \ & \mathrm{tr}\left[(\lambda\mathcal{L}_\mu^{(1)} + (1-\lambda)\mathcal{L}_\mu^{(2)})^2\right] \\
& - 2\mathrm{tr}\left[(I_n - W\Lambda W^T)(\lambda\mathcal{L}_\mu^{(1)} + (1-\lambda)\mathcal{L}_\mu^{(2)})\right]
\end{aligned} \tag{3}$$

  Define

$$\begin{aligned}
a &= \mathrm{tr}\left[(\mathcal{L}_\mu^{(1)})^2 - 2\mathcal{L}_\mu^{(1)}\mathcal{L}_\mu^{(2)} + (\mathcal{L}_\mu^{(2)})^2\right] = \|\mathcal{L}_\mu^{(1)} - \mathcal{L}_\mu^{(2)}\|_F^2 \geq 0. \\
b &= 2\mathrm{tr}\left[(\mathcal{L}_\mu^{(2)} - I_n + W\Lambda W^T)(\mathcal{L}_\mu^{(1)} - \mathcal{L}_\mu^{(2)})\right].
\end{aligned}$$

1) If $a > 0$, we can obtain

$$\lambda = \begin{cases} 0, & \text{if } -\frac{b}{2a} < 0 \\ 1, & \text{if } -\frac{b}{2a} > 1 \\ -\frac{b}{2a}, & \text{otherwise} \end{cases}$$

2) If $a = 0$, then we can deduce $b = 0$. In this case, We keep λ unchanged. That means if λ_k represent the value of λ in kth iteration, we will let $\lambda_k = \lambda_{k-1}$.

- **Updating $\boldsymbol{W}$**: Given fixed $\lambda, \Lambda, H, \boldsymbol{\mu}$, the update of $\boldsymbol{W}$ can be generated through the optimization problem below

$$\min_{W^T W = I_c} \operatorname{tr}(\Lambda W^T B W) \tag{4}$$

where $B = \lambda \mathcal{L}_{\mu}^{(1)} + (1-\lambda)\mathcal{L}_{\mu}^{(2)} - \frac{1}{2} H H^T$.

The solution W of Eq.(4) can be calculated as the first c eigenvectors of B.

- **Updating $\boldsymbol{\Lambda}$**: Given fixed $\lambda, W, H, \boldsymbol{\mu}$, we optimize the following problem to update $\boldsymbol{\Lambda}$:

$$\min_{0 \le \Lambda_{ii} \le 1, \Lambda_{ij}=0 (i \neq j)} \operatorname{tr}(\Lambda^2 + 2\Lambda C), \tag{5}$$

where

$$C = W^T \left[(\lambda \mathcal{L}_{\mu}^{(1)} + (1-\lambda)\mathcal{L}_{\mu}^{(2)}) - \frac{1}{2} H H^T \right] W - I_c$$

We can get:

$$\Lambda_{ii} = \begin{cases} 0, & C_{ii} \ge 0 \\ 1, & C_{ii} \le -1 \\ -C_{ii}, & \text{otherwise} \end{cases}$$

- **Updating $\boldsymbol{H}$**: Fixing $\lambda, W, \Lambda, \boldsymbol{\mu}$, the optimization problem with respect to H can be reduced into the following formula

$$\min_{H^T H = I_c} \operatorname{tr}(H^T (I_n - W \Lambda W^T) H) \tag{6}$$

Then we can obtain the solution H of Eq. (6) by calculating the first c eigenvectors of $I_n - W\Lambda W^T$.

- **Updating $\boldsymbol{\mu}$**: Given fixed λ, W, Λ, H, then we can optimize the problem in the following form:

$$\begin{aligned} \min_{\|\boldsymbol{\mu}\|_1 = 1, \boldsymbol{\mu} \ge 0} \operatorname{tr}\Big[& -2(\lambda \mathcal{L}_{\mu}^{(1)} + (1-\lambda)\mathcal{L}_{\mu}^{(2)}) \\ & + 2W\Lambda W^T (\lambda \mathcal{L}_{\mu}^{(1)} + (1-\lambda)\mathcal{L}_{\mu}^{(2)}) \\ & + (\lambda \mathcal{L}_{\mu}^{(1)} + (1-\lambda)\mathcal{L}_{\mu}^{(2)})^2 \Big] + \alpha \boldsymbol{\mu}^T M \boldsymbol{\mu} \end{aligned} \tag{7}$$

The optimization problem can be rewritten as a standard quadratic programming formulation, which can be effectively solved with MATLAB *quadprog*.

The algorithm below presents the process of optimization for better understanding of scHoML.

Algorithm 1 High-order Laplacian Matrix Optimization for single cell multiomics data: scHoML

Input: Datasets:$\{X_1, X_2, \cdots, X_V\}$, dimensionality of common embedding c, number of nearest neighbors k.
Output: Low dimensional Embedding $\boldsymbol{H}$

```
Compute $L_p^{(i)}$ of each modal $X_p$, $p = 1, 2, \ldots, V; i = 1, 2$.
Initialize $\lambda, \boldsymbol{W}, \boldsymbol{\Lambda}, \boldsymbol{\mu}$.
repeat
    Update $\lambda$ by solving optimization problem in Eq.(3).
    Update $\boldsymbol{W}$ by solving Eq.(4).
    Update $\boldsymbol{\Lambda}$ by solving Eq.(5).
    Update $\boldsymbol{H}$ by solving Eq.(6).
    Update $\boldsymbol{\mu}$ by solving Eq.(7).
until converge.
```

Convergence & Complexity

- **Convergence Analysis**
  Since Laplacian matrix is a positive semi-definite matrix, we can conclude that the objective function of scHoML takes zero as lower bound. Obtaining its global optimal solution is difficult, because the objective function is non convex. If alternative optimization framework is applied, the objective function value decreases while updating variables. Therefore, the algorithm will eventually converge to a local solution.

- **Complexity**
  The computational complexity of scHoML is mainly caused by SVD decomposition when updating W and H, and its corresponding complexity is $O(n^3)$. Meanwhile, the complexity of updating λ and Λ is $O(1)$ and $O(n)$, respectively. Furthermore, to update μ, we need to solve a standard quadratic programming problem. Let ε be the precision of the result and V be the number of modals, the complexity of solving the quadratic programming problem is $O(\varepsilon^{-1}V)$. If the algorithm has been run for t iterations, the total complexity of our method is $O(t(n^3 + n + \varepsilon^{-1}V))$. If $\varepsilon^{-1} \ll n^2$, the complexity of scHoML can be considered as $O(tn^3)$.

1.2 Clustering with Inferred Low-dimensional Representation

In the optimization of high-order neighborhood Laplace matrix, we simultaneously obtain a common low dimensional embedding $H \in R^{n\times c}$ for the single cell multi-modal data. The cell sub-populations can be identified from the matrix H through appropriate evaluation on the cellular relationships between cells.

Assume $H = [h_1, h_2, \ldots, h_n]^T \in R^{n\times c}$, we model the distance between cell s and cell t $(s, t = 1, 2, \ldots, n)$ as

$$\text{Dis}(s,t) = 1 - \frac{(h_s - \bar{h}_s)(h_t - \bar{h}_t)^T}{\sqrt{(h_s - \bar{h}_s)(h_s - \bar{h}_s)^T}\sqrt{(h_t - \bar{h}_t)(h_t - \bar{h}_t)^T}}$$

where $\bar{h}_s = \frac{1}{c}\sum_{j=1}^{c} x_{sj}, s = 1, 2, \ldots n.$ and

$$\text{Dis}(s,t) = \sum_{k=1}^{c} |h_s(k) - h_t(k)|.$$

Agglomerative hierarchical clustering was performed on the constructed distance matrix to entangle the heterogeneity embedded in the cells.

An appropriate evaluation of the cluster number is critical. We here provide a grain to coarse design of the optimal cluster number. The cluster number is determined through solving the following optimization problems.

If the involved number of samples is small, we strive to evaluate the sample specific silhouette coefficient to measure the clustering matching degree and optimize the mean silhouette coefficient of all samples to determine the best cluster number $c^{\star}_{no}$:

$$c^{\star}_{no} = \text{argmax}_{k\in\mathcal{K}} \sum_{i=1}^{n} (b_k(i) - a_k(i))/\max(a_k(i), b_k(i))$$

where $a_k(i) = \frac{1}{|C_I|-1}\sum_{j\in C_I, i\neq j} d(i,j)$ is the average distance from the i-th point to the other points in the same cluster I as i, and $b_k(i) = \min_{J\neq I}\frac{1}{|C_J|}\sum_{j\in C_J} d(i,j)$ is the minimum average distance from the i-th point to points in a different cluster J, minimized over clusters. For different cluster number $k \in \mathcal{K}$, we run agglomerative hierarchical clustering to generate different clustering results. If most samples have a high silhouette value, the clustering solution is believed appropriate.

If the involved number of samples is relatively large, we propose statistical measures in terms of variance for evaluation of cluster number appropriateness.

$$c^{\star}_{no} = \text{argmax}_{k\in\mathcal{K}} \frac{\text{tr}(\sum_{q=1}^{k} n_q(c_q - c_E)(c_q - c_E)^T)/(k-1)}{\text{tr}(\sum_{q=1}^{k}\sum_{x\in C_q}(x - c_q)(x - c_q)^T))/(n-k)},$$

where C_q is the set of all data in class q, c_q is the central point of class q, c_E is the central point of all data involved, and n_q is the total number of

data points in class q. It is reasonable that we evaluate inter class variance and intra class variance to determine the optimal cluster number $c_{no}^{\star}$ when $\frac{\mathrm{tr}(\sum_{q=1}^{k} n_q(c_q-c_E)(c_q-c_E)^T)/(k-1)}{\mathrm{tr}(\sum_{q=1}^{k}\sum_{x\in C_q}(x-c_q)(x-c_q)^T))/(n-k)}$ achieves maximum.

2 Figures and Tables

Table 1: Performance comparisons of different methods in terms of ARI.

Algorithm\Dataset	simulation data1	simulation data2	mESC	pbmc_10X	pbmc_inhouse
SCbest	0.2426 ± 0.0233	0.2323 ± 0.0072	0.3764 ± 0.0000	0.7214 ± 0.0411	0.6679 ± 0.0742
MSE	0.2709 ± 0.0050	0.0111 ± 0.0059	0.3764 ± 0.0000	0.7161 ± 0.0133	0.6807 ± 0.0502
CoregSC	0.2529 ± 0.0080	0.1368 ± 0.0280	0.1716 ± 0.0628	0.7162 ± 0.0164	0.5152 ± 0.1351
AASC	0.1755 ± 0.0044	0.0063 ± 0.0089	0.7022 ± 0.0235	0.6805 ± 0.0799	0.6027 ± 0.0290
RMSC	0.0423 ± 0.0094	0.0745 ± 0.0050	0.3468 ± 0.0000	0.5724 ± 0.0001	0.5212 ± 0.0266
AMGL	0.1424 ± 0.0892	-0.0246 ± 0.0123	0.4869 ± 0.4691	0.6332 ± 0.1047	0.7275 ± 0.1452
AWP	0.3338 ± 0.0000	0.1154 ± 0.0000	0.2416 ± 0.0000	0.7230 ± 0.0000	0.5693 ± 0.0000
OPMC	0.0241 ± 0.0463	0.6927 ± 0.3311	0.2269 ± 0.2012	0.6133 ± 0.0648	0.8778 ± 0.1095
scAI-hc	0.9391 ± 0.0000	0.6587 ± 0.0000	0.5480 ± 0.0000	0.5131 ± 0.0000	0.7875 ± 0.0000
scAI-leiden	0.9539 ± 0.0000	0.8042 ± 0.0000	0 ± 0.0000	0.0738 ± 0.0000	0.3317 ± 0.0000
scHoML	0.9854 ± 0.0000	1 ± 0.0000	0.9350 ± 0.0000	0.8737 ± 0.0000	0.9523 ± 0.0000

Table 2: Performance comparisons of different methods in terms of NMI.

Algorithm\Dataset	simulation data1	simulation data2	mESC	pbmc_10X	pbmc_inhouse
SCbest	0.2630 ± 0.0149	0.2572 ± 0.0047	0.3894 ± 0.0000	0.8144 ± 0.0070	0.8456 ± 0.0470
MSE	0.3196 ± 0.0098	0.0404 ± 0.0108	0.3894 ± 0.0000	0.8149 ± 0.0004	0.8536 ± 0.0325
CoregSC	0.2796 ± 0.0026	0.450 ± 0.0295	0.2742 ± 0.0341	0.8125 ± 0.0057	0.6456 ± 0.1145
AASC	0.2456 ± 0.0027	0.0592 ± 0.0120	0.6198 ± 0.0200	0.7374 ± 0.0398	0.6877 ± 0.0195
RMSC	0.1130 ± 0.0295	0.0688 ± 0.0027	0.3719 ± 0.0000	0.6705 ± 0.0000	0.6179 ± 0.0186
AMGL	0.2313 ± 0.0787	0.1140 ± 0.0231	0.5166 ± 0.4328	0.7106 ± 0.0574	0.8615 ± 0.0701
AWP	0.3704 ± 0.0000	0.1703 ± 0.0000	0.3122 ± 0.0000	0.8078 ± 0.0000	0.7225 ± 0.0000
OPMC	0.0329 ± 0.0450	0.7343 ± 0.3040	0.2372 ± 0.1989	0.6939 ± 0.0149	0.8815 ± 0.0298
scAI-hc	0.9112 ± 0.0000	0.7892 ± 0.0000	0.5003 ± 0.0000	0.6232 ± 0.0000	0.7906 ± 0.0000
scAI-leiden	0.9348 ± 0.0000	0.8080 ± 0.0000	0.3233 ± 0.0000	0.5128 ± 0.0000	0.7170 ± 0.0000
scHoML	0.9764 ± 0.0000	1 ± 0.0000	0.8729 ± 0.0000	0.8374 ± 0.0000	0.9470 ± 0.0000

Table 3: Performance comparisons of different resolution parameters in scAI-leiden for considered datasets.

Dataset\Resolution	0.01	0.1	0.2	0.3	0.4	0.5
simulation_data1	0/0.4548	**0.9539/0.9348**	0.7021/0.7779	0.7062/0.7886	0.7051/0.7882	0.5474/0.7359
simulation_data2	0.5957/0.7457	**0.8042/0.8080**	0.5587/0.7225	0.3912/0.6746	0.3531/0.6631	0.3167/0.6459
mESC	0/0.3233	0/0.3233	**0.3468/0.3719**	0.3468/0.3719	0.3468/0.3719	0.2182/0.2993
pbmc_inhouse	**0.7215/0.8638**	0.3317/0.7170	0.2327/0.6722	0.1804/0.6457	0.1573/0.6315	0.1429/0.6218
pbmc_10X	**0.2749/0.5996**	0.0738/0.5128	0.0496/0.4934	0.0398/0.4824	0.0348/0.4795	0.0296/0.4722
Dataset\Resolution	0.6	0.7	0.8	0.9	1	
simulation_data1	0.5474/0.7359	0.5425/0.7349	0.5348/0.7298	0.5093/0.7148	0/0.4548	
simulation_data2	0.2921/0.6349	0.2714/0.6266	0.2484/0.6106	0.2283/0.6128	0/0.4630	
mESC	0.2182/0.2933	0.2106/0.3145	0.2727/0.3224	0.1596/0.3020	0/0.3233	
pbmc_inhouse	0.1211/0.6085	0.1066/0.5958	0.1044/0.5939	0.0944/0.5851	0/0.4394	
pbmc_10X	0.0272/0.4687	0.0239/0.4635	0.0208/0.4600	0.0180/0.4543	0/0.4186	

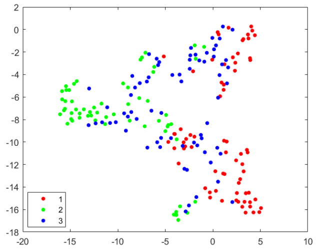

(a) AASC

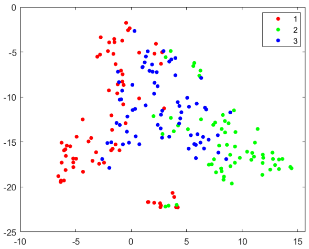

(b) AMGL

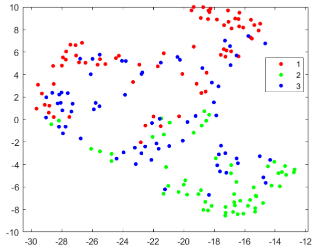

(c) MSE

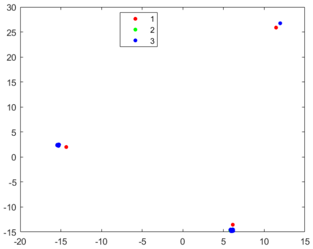

(d) OPMC

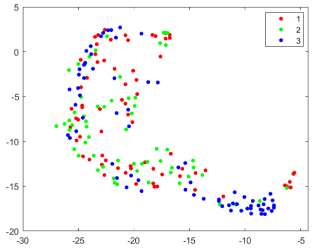

(e) RMSC

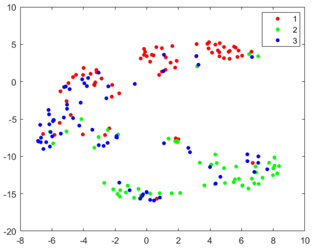

(f) SCbest

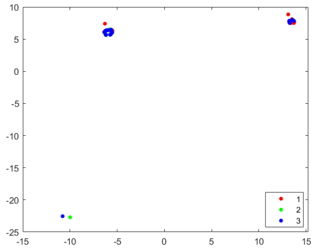

(g) AWP

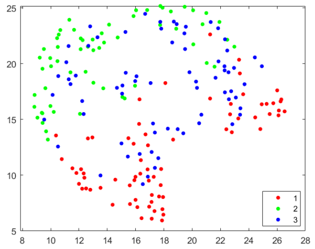

(h) CoregSC

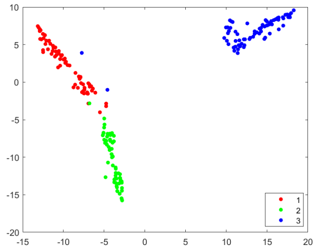

(i) scAI

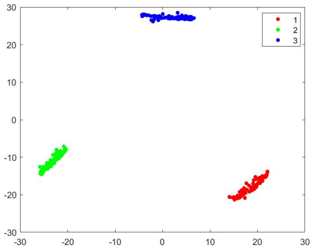

(j) scHoML

Figure 1: tsne plots of different methods in obtaining low-dimensional embeddings for simulation data1

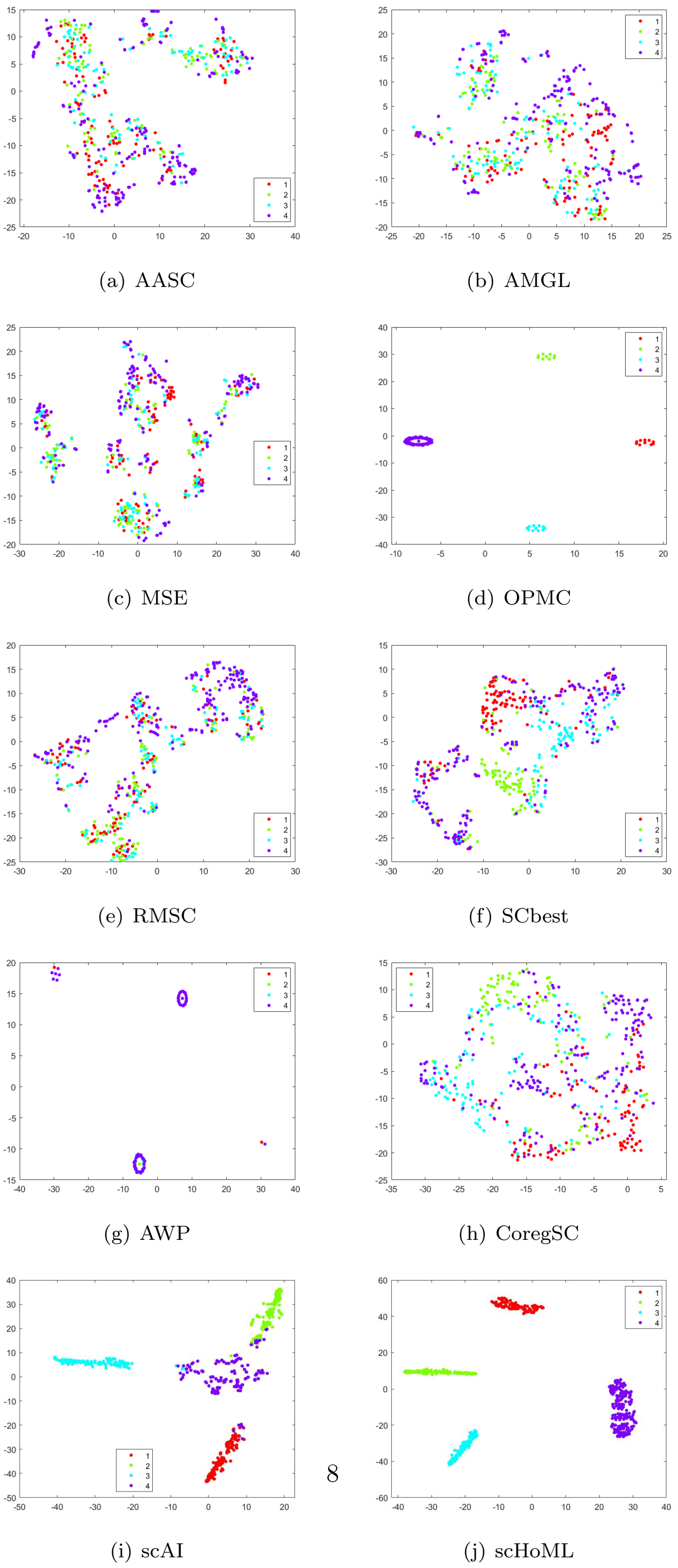

(a) AASC (b) AMGL

(c) MSE (d) OPMC

(e) RMSC (f) SCbest

(g) AWP (h) CoregSC

(i) scAI (j) scHoML

Figure 2: tsne plots of different methods in obtaining low-dimensional embeddings for simulation data2

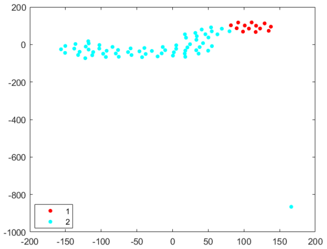

(a) AASC

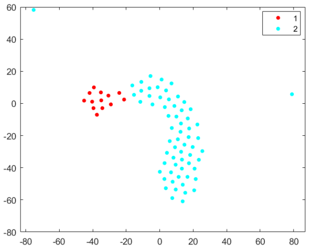

(b) AMGL

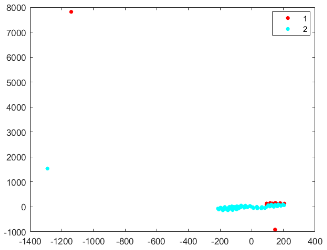

(c) MSE

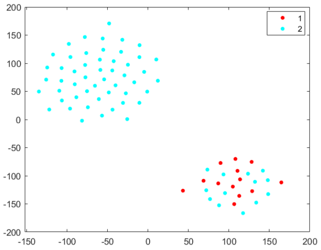

(d) OPMC

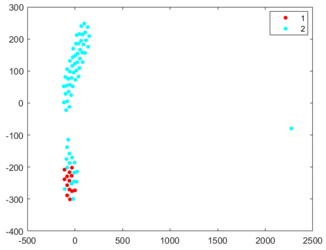

(e) RMSC

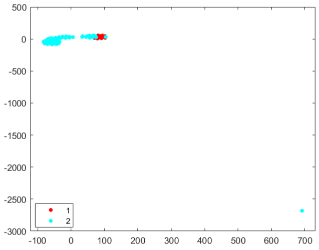

(f) SCbest

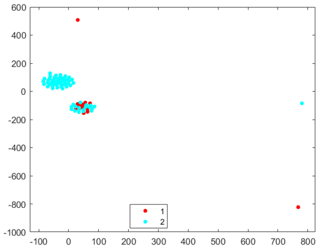

(g) AWP

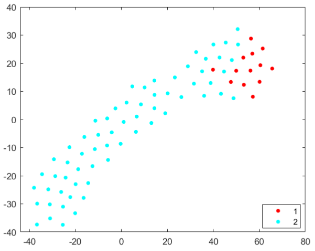

(h) CoregSC

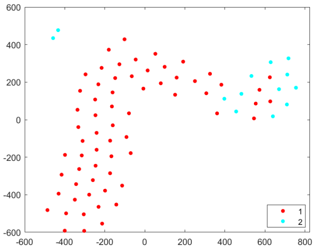

(i) scAI

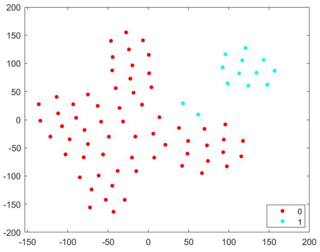

(j) scHoML

Figure 3: tsne plots of different methods in obtaining low-dimensional embeddings for mESC data

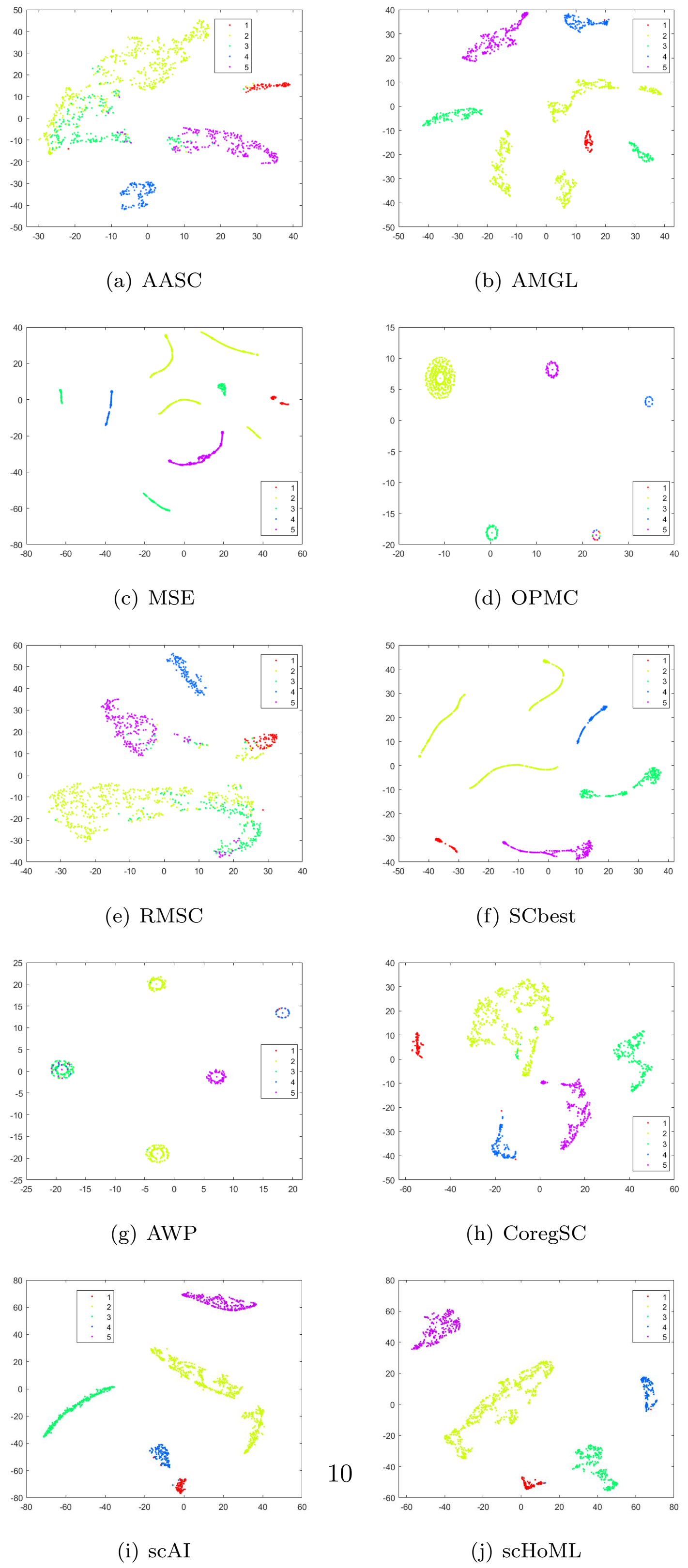

(a) AASC (b) AMGL

(c) MSE (d) OPMC

(e) RMSC (f) SCbest

(g) AWP (h) CoregSC

(i) scAI (j) scHoML

Figure 4: tsne plots of different methods in obtaining low-dimensional embeddings for pbmc_inhouse data

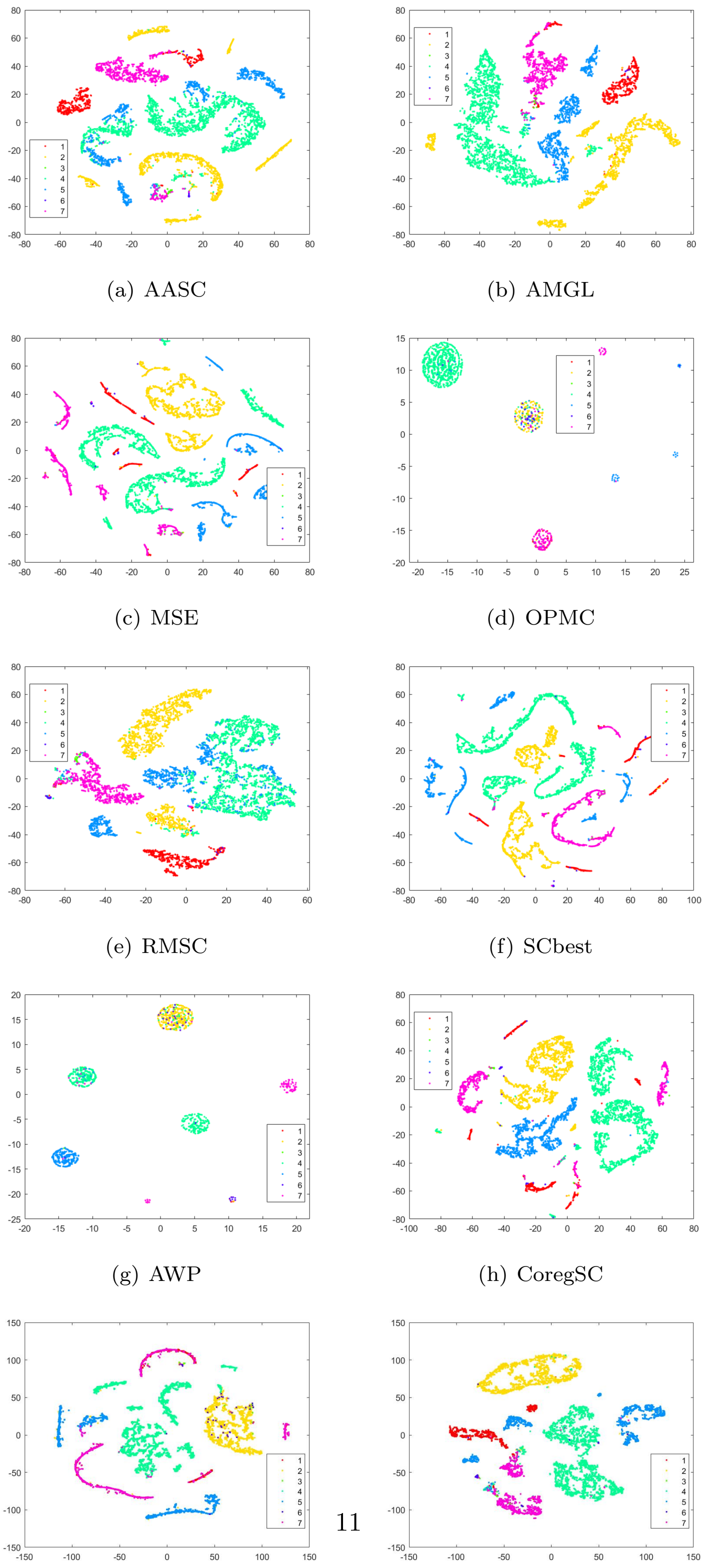

(a) AASC (b) AMGL

(c) MSE (d) OPMC

(e) RMSC (f) SCbest

(g) AWP (h) CoregSC

(i) scAI (j) scHoML

Figure 5: tsne plots of different methods in obtaining low-dimensional embeddings for pbmc_10X data

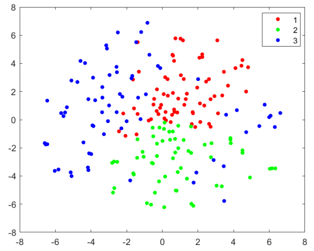

(a) simulation data1-scRNA-seq

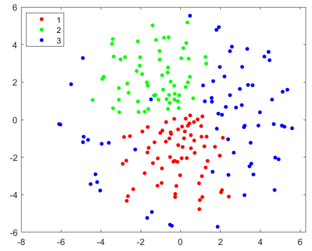

(b) simulation data1-scATAC-seq

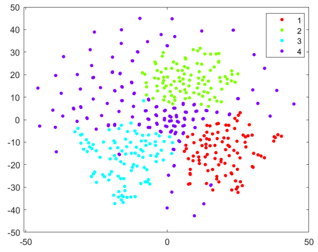

(c) simulation data2-scRNA-seq

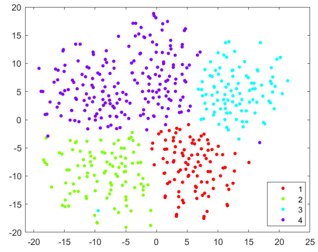

(d) simulation data2-scATAC-seq

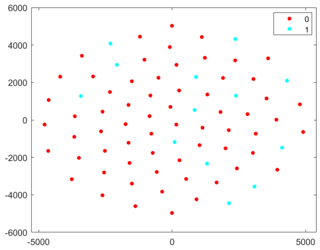

(e) mESC-DNA

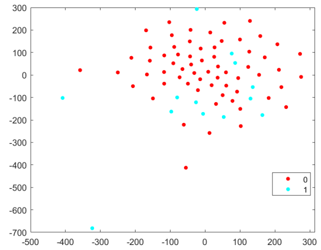

(f) mESC-RNA

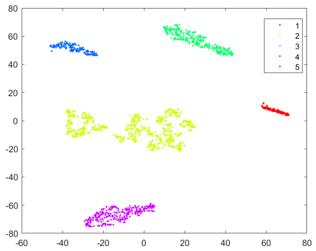

(g) pbmc_inhouse-ADT

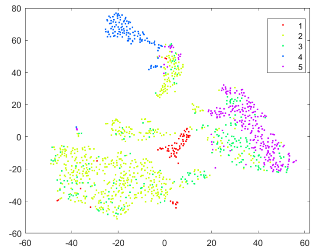

(h) pbmc_inhouse-RNA

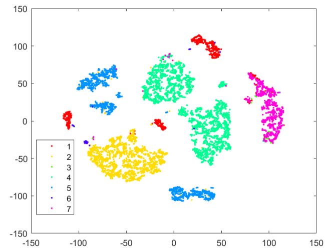

(i) pbmc_10X-ADT

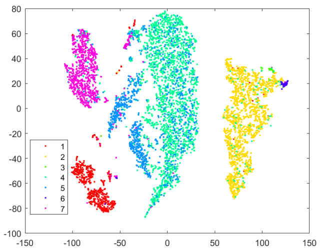

(j) pbmc_10X-RNA

Figure 6: tsne plots for original multi-omics data

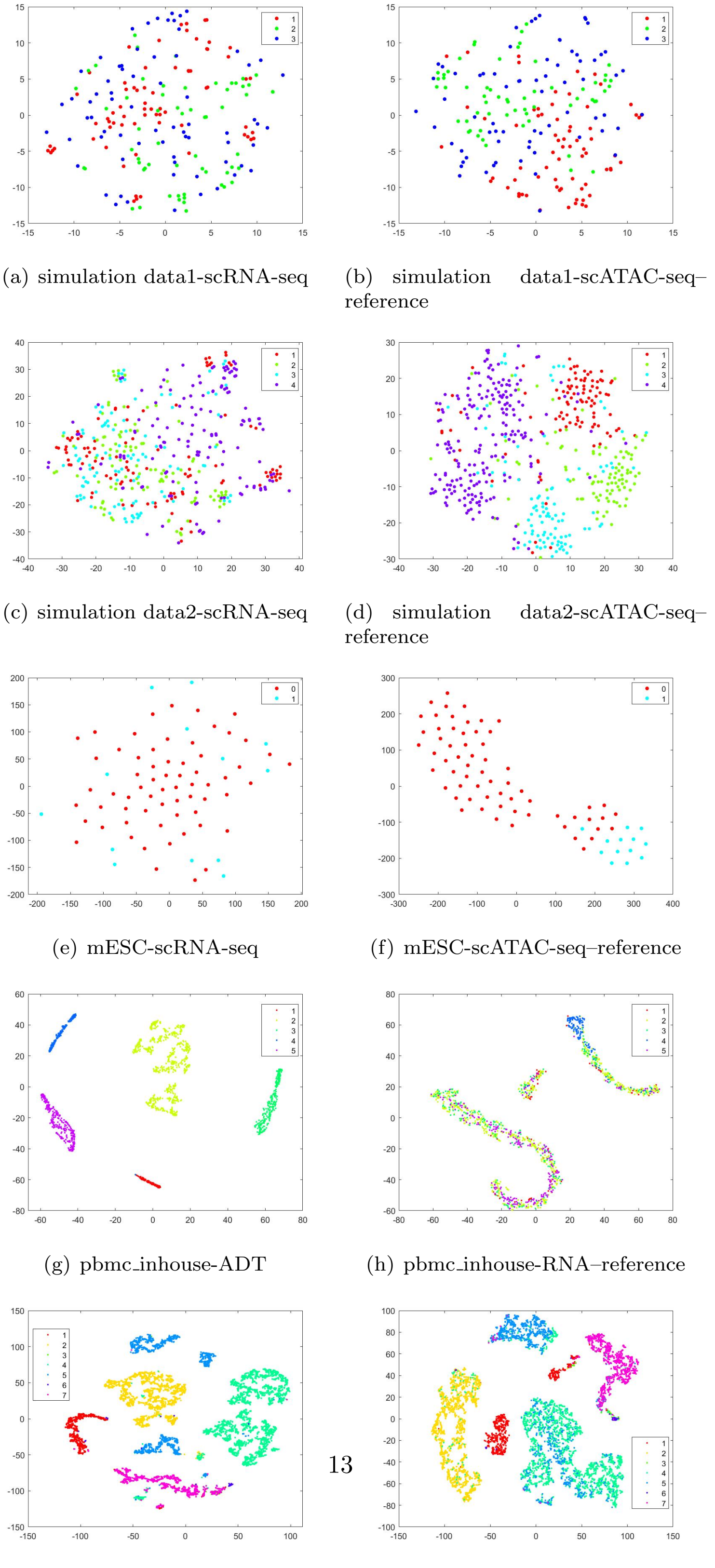

(a) simulation data1-scRNA-seq

(b) simulation data1-scATAC-seq–reference

(c) simulation data2-scRNA-seq

(d) simulation data2-scATAC-seq–reference

(e) mESC-scRNA-seq

(f) mESC-scATAC-seq–reference

(g) pbmc_inhouse-ADT

(h) pbmc_inhouse-RNA–reference

(i) pbmc_10X-ADT

(j) pbmc_10X-RNA–reference

Figure 7: tsne plots for embedding data by UnionCom

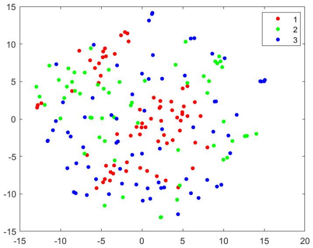

(a) simulation data1-scRNA-seq–reference

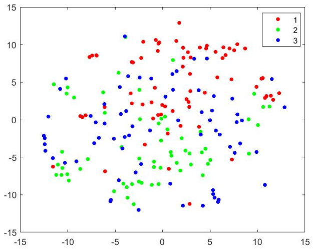

(b) simulation data1-scATAC-seq

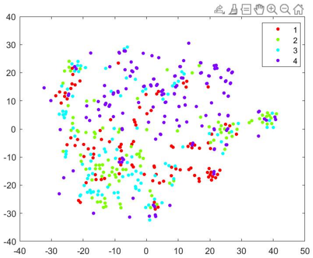

(c) simulation data2-scRNA-seq–reference

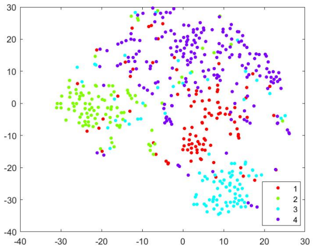

(d) simulation data2-scATAC-seq

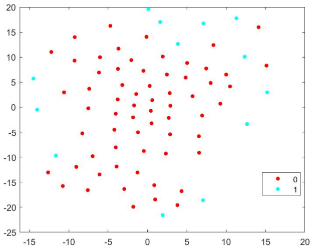

(e) mESC-scRNA-seq–reference

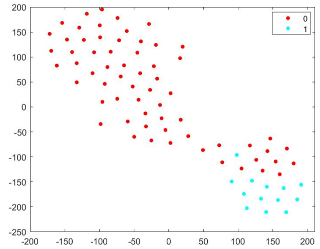

(f) mESC-scATAC-seq

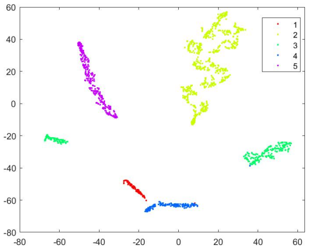

(g) pbmc_inhouse-ADT–reference

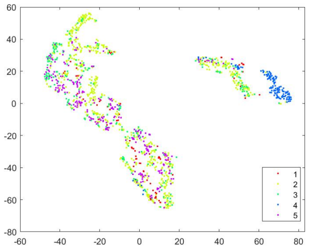

(h) pbmc_inhouse-RNA

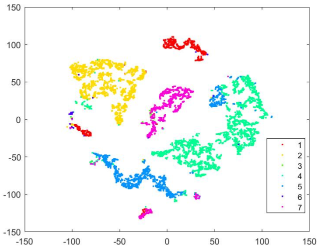

(i) pbmc_10X-ADT–reference

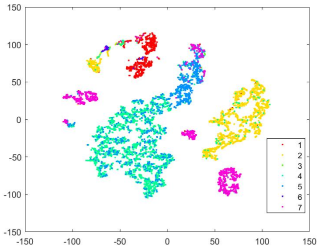

(j) pbmc_10X-RNA

Figure 8: tsne plots for embedding data by UnionCom
